# Liquid BIOpsy for MiNimal RESidual DiSease Detection in Head and Neck Squamous Cell Carcinoma (LIONESS)—a personalised circulating tumour DNA analysis in head and neck squamous cell carcinoma

**DOI:** 10.1038/s41416-022-01716-7

**Published:** 2022-02-07

**Authors:** Susanne Flach, Karen Howarth, Sophie Hackinger, Christodoulos Pipinikas, Pete Ellis, Kirsten McLay, Giovanni Marsico, Tim Forshew, Christoph Walz, Christoph A. Reichel, Olivier Gires, Martin Canis, Philipp Baumeister

**Affiliations:** 1 Department of Otorhinolaryngology, Head and Neck Surgery, Hospital of the Ludwig-Maximilians-University (LMU) of Munich, Marchioninistrasse 15, 81377 Munich, Germany; 2 Inivata Ltd, Babraham Research Park, Cambridge, UK; 3 Institute of Pathology, Faculty of Medicine, LMU Munich, Munich, Germany; 4 Clinical Cooperation Group “Personalised Radiotherapy in Head and Neck Cancer”, German Research Centre for Environmental Health GmbH, Neuherberg, Munich, Germany

**Keywords:** Head and neck cancer, Tumour biomarkers, Translational research, Surgical oncology

## Abstract

**Background:**

Head and neck squamous cell carcinoma (HNSCC) remain a substantial burden to global health. Cell-free circulating tumour DNA (ctDNA) is an emerging biomarker but has not been studied sufficiently in HNSCC.

**Methods:**

We conducted a single-centre prospective cohort study to investigate ctDNA in patients with p16-negative HNSCC who received curative-intent primary surgical treatment. Whole-exome sequencing was performed on formalin-fixed paraffin-embedded (FFPE) tumour tissue. We utilised RaDaR^TM^, a highly sensitive personalised assay using deep sequencing for tumour-specific variants, to analyse serial pre- and post-operative plasma samples for evidence of minimal residual disease and recurrence.

**Results:**

In 17 patients analysed, personalised panels were designed to detect 34 to 52 somatic variants. Data show ctDNA detection in baseline samples taken prior to surgery in 17 of 17 patients. In post-surgery samples, ctDNA could be detected at levels as low as 0.0006% variant allele frequency. In all cases with clinical recurrence to date, ctDNA was detected prior to progression, with lead times ranging from 108 to 253 days.

**Conclusions:**

This study illustrates the potential of ctDNA as a biomarker for detecting minimal residual disease and recurrence in HNSCC and demonstrates the feasibility of personalised ctDNA assays for the detection of disease prior to clinical recurrence.

## Background

Despite improvements in treatments for squamous cell carcinoma of the head and neck (HNSCC), many patients develop disease recurrence [1]. Fewer than 50% of patients will survive beyond five years. Standard therapy for locally advanced HNSCC involves surgical resection of the primary tumour and regional lymph node metastases, radiotherapy with or without chemotherapy or a combination of modalities. A distinct staging for p16-positive HNSCC as determined by immunostaining, a widely used clinical biomarker for infection with human papillomavirus (HPV), has recently been introduced due to its strong prognostic value in patients with squamous cell carcinomas (SCC) of the oropharynx [2, 3]. Around 30–35% of oropharyngeal tumours are attributable to HPV and are also linked to a significantly better outcome compared to patients diagnosed with HPV-negative, i.e. p16-negative, disease [4]. Unlike other cancers, reliable biomarkers for therapy planning and to monitor treatment response in patients with p16-negative HNSCC do not exist [5]. Instead, initial diagnosis, as well as monitoring of HNSCC, are based solely on clinical findings and imaging with known sensitivity and specificity caveats.

The detection of circulating cell-free tumour DNA (ctDNA) as a marker of minimal residual disease following curative-intent surgery holds promise for identifying patients at an increased risk of relapse, who may benefit from adjuvant radio(chemo)therapy or facilitate close monitoring with repeat resection, if needed. The main advantage of ctDNA is its availability from liquid biopsies that are minimally invasive and easily obtainable throughout the course of the disease, including before any other diagnostic and/or therapeutic measures. Together with the increased cost-effectiveness of next-generation sequencing (NGS), even the smallest amounts of ctDNA can potentially be detected and quantified [6–8]. Over the past decade, several studies have demonstrated the potential applications of ctDNA analysis for early detection of relapse, treatment selection and response monitoring, as well as tracking of tumour heterogeneity in a variety of cancer types including non-small cell lung cancer [9, 10], colon cancer [11, 12], breast cancer [6, 13–15] and others [7, 16–18]. For the subset of HPV-associated oropharyngeal SCC, circulating tumour HPV DNA (ctHPVDNA) has emerged as a promising biomarker for monitoring treatment response and recurrence [19–26]. In contrast, except for a few published studies, ctDNA in HPV-negative HNSCC has not yet been sufficiently studied [27–34]. The heterogenous nature of the disease, including the paucity of activating mutations in oncogenes and predominance of a variety of inactivating genetic alterations in tumour suppressors, have posed barriers to biomarker as well as targeted therapy development [35–40].

For this study, we prospectively collected plasma samples from 17 patients with p16-negative HNSCC primarily treated with curative-intent surgery. The aims of this study were firstly to determine whether post-operative ctDNA detection can act as a biomarker for surgical tumour clearance. Secondly, we wanted to evaluate the potential of personalised ctDNA analysis for early molecular-level detection of relapse prior to clinically confirmed recurrence with concomitant implications for therapy planning. We used a highly sensitive personalised assay design (RaDaR^TM^, Inivata Ltd, Cambridge, UK) to detect ctDNA pre- and post-operatively. Here we demonstrate subsequent detection and longitudinal monitoring of ctDNA following surgery, including detection of molecular relapse ahead of clinical relapse in five of five patients who progressed, and in none of the patients without clinical recurrence.

## Methods

### Study design and patient cohort

LIONESS is a single-centre non-interventional prospective experimental evidence-generating cohort study. Patients with HNSCC of the oral cavity, pharynx or larynx with Stages III-IVb (AJCC 8th edition), p16-negative, deemed resectable and scheduled for surgery were considered eligible. Patients with distant metastasis (cM1) or other active malignancies at the time of enrolment were excluded. Seventeen patients were recruited at the Department of Otorhinolaryngology, Head and Neck Surgery at the Hospital of the Ludwig-Maximilians-University of Munich between April 2020 and April 2021, with a median follow-up of 371 days (292–532 days). As suggested by the local multidisciplinary tumour board, patients received adjuvant radio(chemo)therapy according to the National Comprehensive Cancer Network guidelines (NCCN Guidelines) [41], if necessary. All patients were staged to exclude distant metastasis with computed tomography (CT) and/or magnetic resonance imaging (MRI). Immunohistochemical staining for p16 was done as part of the routine histopathological work-up. Follow-up has been conducted as part of the clinical routine. Pre- and post-operative blood sampling was performed in addition to routine clinical and laboratory examinations. The primary objective was the early identification of patients with minimal residual disease post-operatively and/or molecular-level disease recurrence within 6 months of follow-up with outcome measure determined as presence of ctDNA (i.e., presence of variants measured through RaDaR^TM^) in plasma of patients with HNSCC.

### Plasma sample collection and DNA extraction

Serial plasma samples were collected from 17 patients 1–4 days pre-operatively as well as 2–7 days post-operatively. Additional blood samples were obtained prior to and following adjuvant therapy, if indicated, as well as during follow-up visits. In case of a resectable recurrence, samples were obtained again pre- and post-operatively as mentioned above. Venous blood from study participants was collected by standard phlebotomy techniques in two cell-free DNA blood collection tubes (Streck, La Vista, NE, USA) and sent for further processing to Inivata Ltd (Cambridge, UK). Plasma samples were prepared from up to 10 ml of venous blood, as soon as possible after collection and within 7 days, with an initial centrifugation at 1600 × *g* for 10 min and second centrifugation of plasma aliquots at 20,000 × *g* for 10 min. The buffy coat layer was separated during blood processing and stored at −80 °C. DNA was extracted from 200 µl of buffy coat (leukocytes) using QIAamp DNA Blood Mini Kit (Qiagen) and circulating cell-free DNA (cfDNA) extracted using the QIAamp Circulating Nucleic Acid Kit (Qiagen). Buffy coat and cfDNA were quantified using digital PCR (BioRad QX200) as described previously [42].

### Tumour/germline whole-exome sequencing

FFPE tumour blocks of the resected specimen were obtained and ten 10-µm unstained slides and one haematoxylin and eosin-stained slide were cut from representative FFPE blocks. An experienced head and neck pathologist assessed tumour content and cellularity and suitable tumour areas were marked for macrodissection, if necessary. Tumour tissue slides were sent for whole-exome sequencing (WES) to Inivata Ltd. DNA was extracted from the provided FFPE tumour tissue samples using the Maxwell RSC DNA FFPE kits. The extracted DNA was processed with the KAPA Hyper Prep Kit and indexed uniquely. The resulting pre-capture libraries were quantified using the Quant-iT dsDNA High Sensitivity assay. Each library proceeded to exome enrichment and was analysed on a fragment analyser and quantified using the Quant-iT dsDNA High Sensitivity assay. Sequencing was performed on the HiSeq4000 platform (Illumina).

### Bioinformatics analysis for whole-exome sequencing

The analysis of tumour-only exome-sequencing data was performed using a proprietary pipeline: samples with a median cellularity of 50% (range 40–70%, as estimated by pathology reports) were processed through fastq files processing, alignment, and variant calling. Germline variants were filtered out using custom filters that take into account prior knowledge available from public single nucleotide polymorphism (SNP) databases and variants were filtered based on multiple parameters including allele frequency and depth. Performance characteristics of the WES is shown in Supplementary Table 1.

### RaDaR^TM^ patient-specific assay design

RaDaR^TM^ is a personalised ctDNA assay built on the InVision^®^ platform [43], which utilises multiplex PCR and targeted NGS. Somatic variants identified in the tumour tissue by WES were prioritised using proprietary algorithms to build a patient-specific primer panel of up to 48 primer pairs capturing at least one somatic variant (Supplementary Table 2). The personalised primer panel was manufactured (IDT, Coralville, IA) and combined with a fixed primer panel of 21 common population-specific SNPs for quality control purposes during the NGS testing. An aliquot of tumour DNA from FFPE tissue was used for primer panel qualification to confirm the accuracy and performance of the RaDaR^TM^ panel design.

Following primer panel qualification, RaDaR^TM^ assays were performed on cfDNA from plasma aliquots alongside a buffy coat DNA control sample, which was used for identification and removal of germline variants, the removal of variants due to clonal haematopoiesis of indeterminate potential (CHIP) from the analysis, and as a positive amplification control. Multiplex PCR was performed with input concentrations ranging from 1452 to 20,000 amplifiable copies per sample, as measured by droplet digital PCR, with a median value of 14,550 copies.

### RaDaR^TM^ sequencing analysis

RaDaR^TM^ libraries were sequenced using the Nova-Seq 6000 system (Illumina Inc., San Diego, USA) and sequencing data analysed in a multi-step process: fastq files were demultiplexed using *bcl2fastq*, reads were then aligned using the *bwa mem* alignment software and processed using proprietary software to identify primer pairs and count mutant and reference bases.

Individual variants were subject to quality control in the process of calling residual disease positive or negative. Variants present in the buffy coat material or absent in the tumour tissue DNA were excluded from further analysis. Proprietary methods were used to call residual and recurrent disease: a statistical model was used to assess the statistical significance of the observed mutant counts for each variant and the information was integrated over the entire set of personalised variants to obtain evidence of tumour presence or absence at the sample level. This includes an assessment of the noise of each individual variant class and the sensitivity and specificity based on the number of variants in the panel. A sample will be called as positive for residual disease if its cumulative statistical score is above a pre-set threshold, as defined during analytical development (Supplementary Methods). The tumour fraction estimated from this model was then reported (estimated variant allele frequency, VAF).

### Longitudinal plots of ctDNA detection levels and lead time analysis

A time course of ctDNA detection levels was plotted from the date of pre-operative sample collection. Red dots indicate ctDNA-positive samples, and black dots ctDNA negative samples. Progression is indicated with a yellow triangle, based on annotated clinical recurrence. ctDNA levels are estimated as part of the calling procedure. ctDNA negative samples were assigned a VAF of not detected (ND) and plotted at the bottom of the plot. Lead time was taken to be the interval between detection of the first ctDNA-positive sample post surgery and confirmation of clinical recurrence.

Survival analysis was plotted using a Kaplan Meier Curve, although the limited sample number only allowed qualitative assessment. Each curve represents a specific cohort stratified according to specific criteria—ctDNA detection yes/no—and should approach the true survival function for the population under investigation, provided the sample size is large enough. Each vertical drop in the curve represents an event occurring, in this case relapse: for instance, for the ctDNA-positive patients (red curve), each drop identifies one patient recurring as detected by clinical investigations.

## Results

### Patient characteristics

Seventeen patients with resectable disease at the time of diagnosis were recruited into the LIONESS study and underwent longitudinal blood sampling. 103 serial plasma samples were assessed for evidence of ctDNA (Supplementary Table 3). All 17 patients had blood samples taken before surgery. The median age of the study participants was 63 years (range 48–78 years), and the majority (76.5%) were male, as is typical of HNSCC patients. In our cohort, all patients had either Stage III or IVa-b disease. 2/17 patients had recurrent disease and/or a second primary cancer of the head and neck at diagnosis/enrolment. All patients were scheduled for curative-intent surgery and 15/17 patients received adjuvant treatment according to the recommendations of the multidisciplinary tumour board. One patient declined adjuvant radiotherapy. Another patient was not eligible for further radiotherapy treatment due to previous radiation therapy of the head and neck. The demographics and clinical characteristics of the patient cohort are shown in Table 1. All patients had sufficient tumour tissue available for the WES analysis that was necessary to design the personalised ctDNA assay. In total, 20 tumour regions from 18 tumours obtained from 17 patients were sequenced. Among these, one patient had two synchronous primary tumours in the head and neck area: one laryngeal tumour and one tumour of the floor of the mouth. The latter had to be sequenced twice due to insufficient tumour content at the first attempt. Another patient’s tumour had to be re-sequenced from material obtained from a different area due to initial insufficient tumour cellularity.

**Table 1:** Demographics and clinical data of the patient cohort.

	Absolute number, *n*	%
Age (years)		
Stage III	Median: 67 (range: 55–78)	
Stage IV	Median: 60 (range: 48–76)	
Sex		
Male	13	76.5
Female	4	23.5
Smoking status		
Smoker	8	47.1
Ex-smoker	8	47.1
Never	1	5.8
Location		
Oral cavity	5	27.8
Oropharynx	2	11.1
Larynx	7	38.9
Hypopharynx	4	22.2
Second primary tumour	1	5.9
pT stage		
pT1	2	11.1
pT2	0	0
pT3	12	66.7
pT4	4	22.2
pN stage		
pN0	10	55.5
pN1	1	5.6
pN2	5	27.8
pN3	2	11.1

### ctDNA in pre- and post-operative plasma samples

**Fig. 1:**
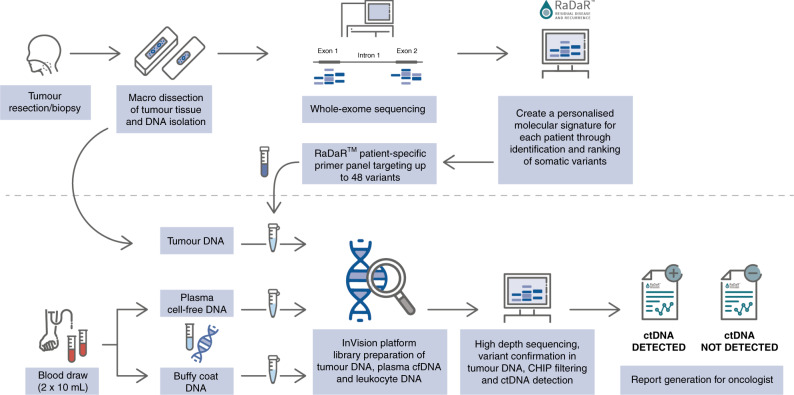
RaDaR^TM^ workflow. Tumour tissue from surgical resection was macrodissected and used for whole-exome sequencing to identify somatic mutations. A personalised ctDNA assay was developed for each patient. Tumour and buffy coat DNA were analysed using personalised assays to confirm somatic mutations and exclude clonal haematopoiesis of indeterminate potential (CHIP). Plasma samples were analysed using RaDaR^TM^ panels and high-depth sequencing and ctDNA detection reported per patient.

**Fig. 2:**
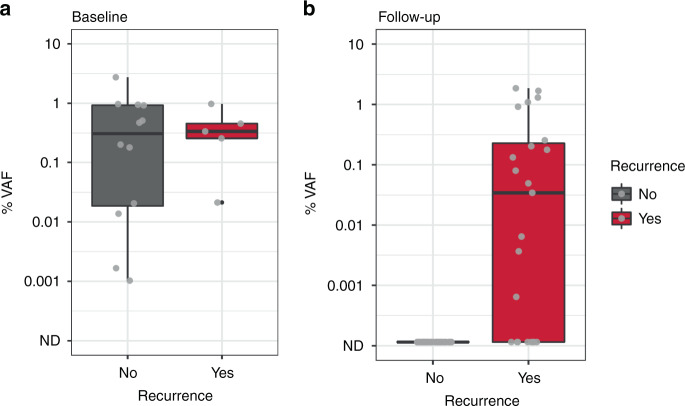
Box plots of estimated median variant allele frequency percentage (% VAF). **a** ctDNA levels in baseline samples taken prior to surgery ranged from 0.001% to 2.737% estimated variant allele frequency (%VAF). **b** In post-surgery samples, ctDNA could be detected at levels as low as 0.0006% VAF, with levels below 0.01% VAF in 20% of ctDNA-positive samples.

**Fig. 3:**
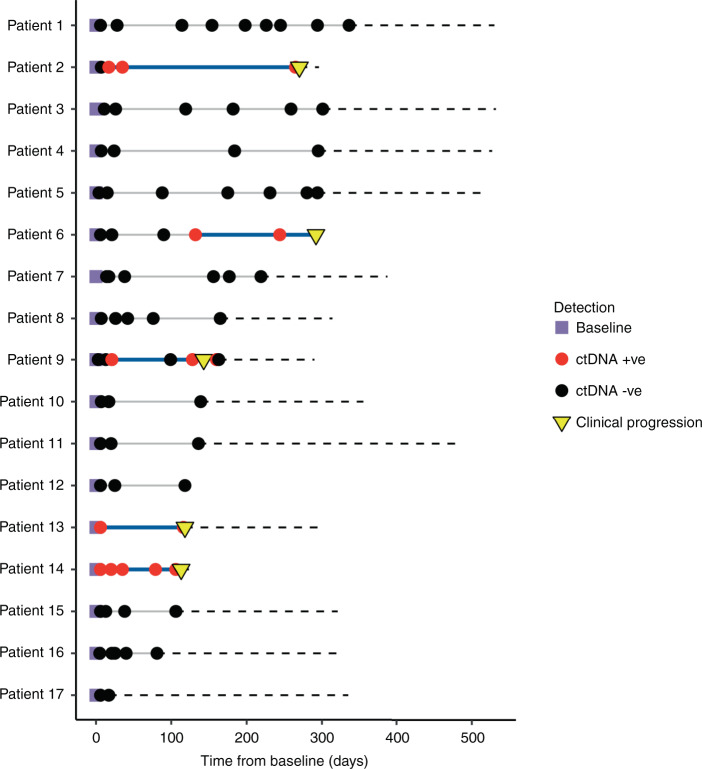
Longitudinal monitoring of serial plasma samples. Longitudinal monitoring of serial plasma samples from 17 patients, indicating when ctDNA was detected (red circle) or not detected (black circle) and whether the patient subsequently relapsed (inverted yellow triangle). ctDNA was detected in all pre-operative time points (purple square). The black dashed line extends across the *x* axis to the last clinical visit to date to indicate the total duration of follow-up for each patient. Patients were followed up for a median duration of 371 days (292–532 days) in the overall cohort, excluding one patient lost to follow-up (patient 12) and four deceased patients (2, 9, 13 and 14). Clinical or CT-morphological evidence of disease recurrence was not observed at the time of last follow-up visit in 12/17 cases profiled. The solid blue line indicates the lead time which is the interval between the first ctDNA-positive post-surgery sample and clinical confirmation of disease recurrence.

**Fig. 4:**
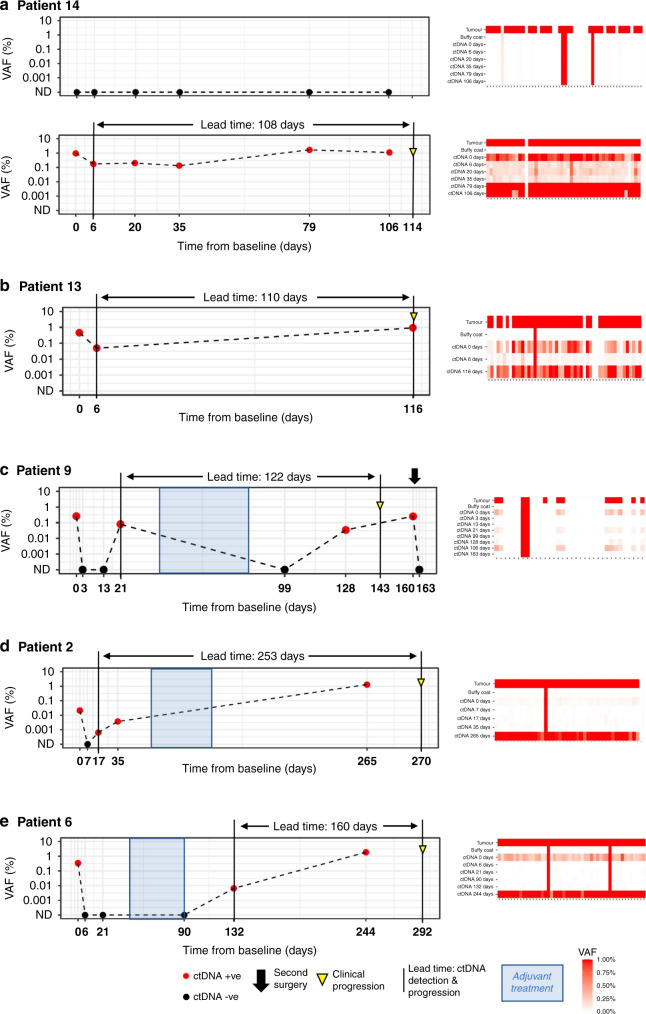
Examples of longitudinal monitoring of ctDNA in five patients. ctDNA detection is indicated with red circles, not detected (ND) with black circles, clinical progression with an inverted yellow triangle and lead time from ctDNA detection to clinical progression with black lines. Periods of adjuvant treatment are shaded in blue. Baseline (time point 0 on the *x* axis) is the pre-surgical plasma collection time point. **a** Patient 14—ctDNA was not detected in the pT1 tumour at any time point for this patient (top panel) but was detected at all time points pre- and post-operatively in the pT4a tumour (bottom panel), with a lead time of 108 days from ctDNA detection to clinical progression. **b** Patient 13—ctDNA detected at all time points, 110 days prior to progression. **c** Patient 9—ctDNA detected before surgery, but not 3 or 13 days post surgery. ctDNA detected at 21 days post surgery, which decreased after completion of adjuvant treatment only to rise again by day 128, prior to clinical progression. ctDNA levels were undetectable following a second surgical intervention at 161 days. **d** Patient 2—ctDNA detected before surgery but not at day 7 after surgery. ctDNA levels rose to detectable levels by day 17 and continued to rise >253 days prior to progression. **e** Patient 6—ctDNA detected before surgery but not at 6, 21 and 90 days post surgery, following adjuvant therapy. Rising ctDNA levels were detected from 132 days, 160 days ahead of clinical progression. For all patients, heatmap on the right of the figure shows the signal from different variants. Each column represents a different variant and each row a different sample type. Variants absent in the tumour DNA or present in the buffy coat DNA were excluded from the analysis.

The RaDaR^TM^ assay demonstrated 95% sensitivity at 0.001% median VAF and 100% specificity in analytical validation studies (Supplementary Methods) and was used to analyse serial pre- and post-operative plasma samples for evidence of minimal residual disease and recurrence (Fig. 1). Personalised panels were designed with between 34 and 52 somatic variants (median 48). Tumour-specific variants were detected as ctDNA pre-operatively in 100% of patients. The median VAF of the pre-operative sample for all disease stages was 0.34%. In post-surgery samples, ctDNA could be detected at levels as low as 0.0006% VAF (Fig. 2). In all cases with clinical recurrence to date (5/5), ctDNA was detected prior to progression, with lead times ranging from 108 to 253 days (Figs. 3 and 4 and Supplementary Fig. 1). Hence, the RaDaR^TM^ assay allows the detection of tumour-derived variants in limited amounts of plasma DNA. We consider the following cases as examples of the improved precision in personalised ctDNA analysis.

### ctDNA detection to identify minimal residual disease

Patient 14 was diagnosed with a synchronous pT4a laryngeal and pT1 floor of the mouth SCC (pN0, Stage IVa) on a background of a previous pT2 pN0 floor of the mouth SCC that had been treated surgically and with adjuvant radiotherapy more than a decade ago. Local resection of both tumours was performed with curative intent. The defect was reconstructed with a supraclavicular artery island flap (SCAIF) and bilateral neck dissection was performed. The patient underwent additional surgery a few days later due to positive resection margins in the floor of the mouth as well as developing a fistula, which was treated with another SCAIF. No adjuvant treatment for Stage IVa disease was possible because of radiation doses the patient had received in the past. Personalised panels were designed for each tumour entity, with 47 somatic variants passing all quality control filters for the laryngeal tumour and 35 somatic variants for the floor of the mouth tumour (Fig. 4a). The ctDNA levels of the small pT1 floor of the mouth tumour were undetectable pre-operatively and remained undetectable throughout the patient’s course of management. In contrast, tumour-specific ctDNA from the large pT4a laryngeal tumour was detectable in pre-operative plasma at 0.977% VAF and remained detectable at 0.178% VAF post-operatively (Fig. 4a). Importantly, the increase in ctDNA preceded the local recurrence confirmed by panendoscopy and biopsy of the neopharynx 108 days later. This suggests that occult minimal residual disease of laryngeal cancer remained present following surgery and resulted in recurrence of the disease around three months later.

All mucosal tissue samples taken after tumour resection were clear in intra-operative frozen section analysis. The pathological examination of the resected tumour tissue revealed not only infiltration of the laryngeal cartilage but also an extension into the extra-laryngeal soft tissue. Therefore, minimal residual disease within the pre-laryngeal soft tissue seemed the most likely cause in retrospect.

Patient 13 demonstrated a similar pattern of post-operative minimal residual disease. Following transoral tumour resection of a pT3 pN0 lateral tongue SCC, reconstruction with a submental flap and bilateral neck dissection, the patient declined adjuvant radiotherapy for Stage III disease. For the personalised ctDNA assay design, 43 somatic variants passed all quality control filters. Pre-operatively, ctDNA was detectable at 0.456% VAF and declined to 0.049% VAF at day 3 post surgery, even though resection margins were clear (≥5 mm), as confirmed by intra-operative frozen section analysis. Once again, the rise in ctDNA levels preceded clinically confirmed local relapse 110 days later (Fig. 4b). Due to inoperability, definitive radiochemotherapy was started. Following the subsequent radiological confirmation of disseminated disease, the patient is now at a palliative stage. This case illustrates the potential of personalised ctDNA monitoring for further treatment decisions as disseminated tumour cells may have caused the post-operative ctDNA detection.

Patient 9 had a pT3 pN0 tumour of the lateral tongue, which was resected in addition to removing the cervical lymph nodes. Only ten somatic variants passed all quality control filters for the personalised ctDNA assay due to a high number of germline variants or variants that were absent in the primary tumour DNA at panel quality control. Pre-operative ctDNA was detected at 0.257% VAF and decreased immediately after surgery to undetectable levels (Fig. 4c). Prior to the start of adjuvant radiotherapy for Stage III disease, ctDNA levels started to rise again to 0.079% VAF only 21 days post surgery. Immediately after completing adjuvant treatment ctDNA levels were undetectable, only to rise again to 0.034% VAF one month after radiation ended. A submucosal lesion in the lateral tongue was palpated during follow-up clinical examination and subsequently confirmed histologically to be a local recurrence. The patient had the local tumour recurrence resected and post-operative ctDNA levels dropped again to undetectable levels. The early increase in ctDNA post-operatively and prior to adjuvant therapy suggests minimal residual disease. Again, the use of personalised ctDNA analysis to detect minimal residual disease following tumour resection may have allowed surgeons to consider additional resection, if possible, and/or a timely start of adjuvant treatment due to the 122-day lead time between ctDNA detection and clinical progression.

Finally, patient 2 exhibited a similar pattern of minimal residual disease and rising ctDNA levels ahead of clinical recurrence (Fig. 4d). Following bilateral neck dissection, laryngectomy, and reconstruction with a SCAIF for a pT4a pN3b (Stage IVb) hypopharyngeal tumour, the patient underwent adjuvant radiochemotherapy. A personalised ctDNA assay was designed with 40 somatic variants passing all quality control filters, and pre-operative ctDNA was detected at 0.021% VAF. Post-operatively, ctDNA was undetectable, however, levels started to increase again 10 days later. Unfortunately, no samples could be collected immediately after the completion of adjuvant therapy. Therefore, it is impossible to say whether ctDNA levels remained elevated throughout the adjuvant treatment, or whether there had been a drop after adjuvant radiochemotherapy followed by another increase to 1.307% VAF, similar to the case described previously. Once again, ctDNA increase preceded clinical confirmation of metastatic disease detected by CT 253 days later. Interestingly, however, only a lung lesion had initially been detected by CT and confirmed to be a SCC by biopsy. At this point in time, it was not possible to distinguish a metastatic lesion from the previously treated hypopharyngeal SCC from a second primary lung SCC. For this patient, the surge in ctDNA suggested the occurrence of a metastasis, which was later confirmed by additional scans to be disseminated disease to the lung and bones.

### ctDNA detection to identify recurrence

Patient 6 had a pT3 pN0 (Stage III) laryngeal cancer and underwent laryngectomy and bilateral neck dissection with curative intent. Of the variants included in the personalised ctDNA assay design, 46 somatic variants were selected. Pre-operatively, ctDNA was detected at 0.336% VAF and decreased immediately after surgery to undetectable levels (Fig. 4e). They remained low when analysed again following adjuvant radiotherapy. Strikingly, ctDNA levels showed a subsequent increase to 0.0064% VAF around one month after completion of adjuvant treatment. Importantly, the increase in ctDNA preceded imaging detection of a local recurrence by 160 days. At this stage, the disease was deemed unresectable, primarily because the patient did not qualify for radical surgery and reconstructive measures needed due to extensive comorbidities.

ctDNA was detected for all patients who had a clinical recurrence, as described above and shown in Supplementary Fig. 2.

## Discussion

This study prospectively evaluated the performance of ctDNA monitoring in a cohort of 17 patients with p16-negative HNSCC planned for curative surgery. We utilised RaDaR^TM^, a highly sensitive personalised assay using deep sequencing of up to 48 primer pairs each capturing at least one tumour-specific variant to analyse serial pre- and post-operative plasma samples for evidence of minimal residual disease and recurrence. Detection of ctDNA was shown to be sensitive to levels of 0.0006%, consistent with previous studies utilising RaDaR^TM^ in breast [44] and lung cancer [45].

Previous studies have shown that ctDNA could be measured in the blood of 70% of patients with HNSCC Stage I and II, in 92% with Stages III and IV [27] and in ~70% of patients with metastatic HNSCC [28]. Wang et al. [27] included 32% HPV-positive cases, whereas Bettegowda et al. [28] made no distinction between association with and without HPV-infection even though clinical, epidemiological, histopathological and molecular evidence suggests that these are two distinct disease entities [35, 46–48]. Similarly, higher levels of ctDNA were detected in patients with clinical N2-N3 disease compared to patients with clinical N0-N1 disease [49]. Furthermore, high levels of ctDNA have been shown to correlate with decreased overall survival and tumour stage [28, 29] as well as lymph node metastases [50]. Most approaches for ctDNA monitoring, however, were limited by inadequate sensitivity and specificity. Using the RaDaR^TM^ assay, we were able to detect pre-operative ctDNA in 100% of patients. In all five cases with clinical recurrence to date, ctDNA was detected prior to progression, with lead times ranging from 108 to 253 days.

In five case studies presented here, we demonstrate how ctDNA detection could have been applied to aid therapy planning and clinical decision-making. Depending on the disease stage, reconsideration for proceeding with close clinical monitoring may even be possible, particularly for those patients where the disease was discovered early enough and where adjuvant treatment may be considered optional. Confirmation of post-operative tumour clearance by undetectable ctDNA could potentially allow a more flexible start of additional therapeutic measures. However, interventional studies are needed to evaluate whether ctDNA monitoring could be an additional tool to stratify patients for adjuvant therapy or clinical follow-up.

In contrast to suggested tumour clearance by undetectable ctDNA levels, positive post-operative ctDNA or an increase of ctDNA levels soon after tumour resection may indicate minimal residual disease independently from intra-operative analysis of frozen sections. With this knowledge at hand, the operating surgeon may consider additional resection(s) of the tumour bed or revision neck dissection, particularly in cases where adjuvant treatment will not be possible or plan further surgeries accordingly. In cases where additional operative procedures are not feasible, a timely start of adjuvant treatment should be expedited. It is noteworthy, that when patients decline adjuvant therapy, as has been the case with patient 13, results from a personalised ctDNA assay may offer additional means to persuade an indecisive patient to undergo necessary and potentially life-saving adjuvant radio(chemo)therapy. Having a single blood test done, even at a local GP practice, may also improve compliance with clinical follow-up appointments in this patient cohort where addictive behaviour, including alcohol abuse and smoking, as well as lack of adherence to medical recommendations is frequently experienced [51–53]. Nevertheless, this should be accompanied by close psychological support, particularly in cases where a positive test may precede any potentially confirmed recurrence of the disease.

Furthermore, rising ctDNA levels prior to start of adjuvant treatment may trigger re-assessment of distant lesions initially deemed non-suspicious in pre-operative imaging. CT imaging of patient 2 done pre-operatively, for example, showed several, very small subpleural lesions in the lung, that, retrospectively, could well correspond to the subsequently confirmed disseminated pleural metastases. Differentiating a single pulmonary metastasis from primary lung SCC is of high clinical importance, but not possible in most cases with current diagnostics because these tumours often share the same histomorphology and immunohistochemical profiles [54]. This knowledge, however, would have a significant impact on the choice of treatment strategy: one would be in most cases the initial stage of systemic dissemination that should be treated systemically, the other one would be a potentially curable disease through resection of a second primary tumour. Personalised ctDNA analysis, as has been demonstrated in the case of patient 2, could potentially enable clinicians to make such a significant distinction. Detection of rising ctDNA levels post-operatively may also indicate tumour recurrence, thus requiring an escalation of diagnostic measures such as additional scans. At an earlier time, PET-CT imaging may have enabled clinicians to detect the recurrence at a resectable stage. We demonstrated that RaDaR^TM^ was able to detect ctDNA post-operatively in five cases, preceding the clinical diagnosis of recurrence by many weeks. In most, if not all cases, earlier detection of recurrence may lead to a less radical choice of therapy and may even be the only chance of cure for the patient.

CT scans post-treatment as part of the routine clinical follow-up are commonly performed, helping clinicians to make choices about further clinical management. Here, the evaluation of ambiguous radiological findings may also be aided by personalised ctDNA analysis. For instance in patient 9, a CT scan conducted as a result of a clinically palpable submucosal induration of the tongue was reported to show no sign of local recurrence. Importantly, panendoscopy and biopsy subsequently confirmed the clinical suspicion of local relapse. In retrospect, ctDNA detection at the time of a clinically palpable tongue induration clearly indicated recurrence. Importantly, the MRI scan conducted after histopathological confirmation of a recurrence only showed a diffuse contrast enhancement within the left intrinsic musculature of the tongue even though the tumour was found to be more than 3 cm in diameter (yrpT3) following resection. This observation may suggest that ctDNA analysis offers a unique opportunity to alter patient management, therefore potentially improving survival. In addition, it may also spare patients from unnecessary and risky endoscopic procedures triggered by inconclusive findings of CT and/or PET-CT scans if ctDNA remains undetectable during follow-up. However, further studies are needed to support this.

Interestingly, there is only limited evidence for the effectiveness of follow-up in patients with HNSCC. No international consensus concerning the intervals and duration for follow-up examinations exists, or details about techniques to be used [55]. Currently, a variety of measures such as endoscopic examination, ultrasound scans of the neck, various blood tests, serum tumour markers and imaging studies are being used [56]. In cases of HPV-associated oropharyngeal SCC, the absence of ctHPVDNA has been shown to be associated with recurrence-free survival, whereas detection of ctHPVDNA was not specific for recurrent disease [26]. This suggests a possible application for ctDNA monitoring for recurrence in HPV-positive HNSCC as well. Nevertheless, ctHPVDNA is currently by far the best marker to monitor tumours of this subgroup of HNSCC. The present results suggest that personalised ctDNA analysis may offer a specific and sensitive method for disease monitoring in patients with HPV-negative HNSCC, thus complementing ongoing efforts to find clinically useful markers for HNSCC.

Limitations of this study include the relatively small number of patients (*n* = 17) enrolled to date as well as the still ongoing follow-up with the shortest follow-up length amounting to 10 months. We acknowledge that more patients of this cohort are likely to recur at a later stage, however, using RaDaR^TM^ we were able to detect early molecular-level recurrence within the first 6 months of follow-up. Larger cohort sizes with longer follow-up are needed to allow for sufficiently powered statistical analyses and to correlate ctDNA detection with recurrence and survival. Furthermore, ctDNA detection is reported at the sample level, giving a high degree of sensitivity, but does not inform on the levels and tracking of individual variants or the emergence of sub-clones. In addition, our personalised ctDNA assay design requires suitable FFPE tumour material to be available for WES, which may not always be achievable and may limit the use of this method in certain specific cases. However, a strength of our study includes the use of a personalised ctDNA assay design that is highly sensitive and specific and can be applied to all patients with available WES data. Analytical validation demonstrated that RaDaR^TM^’s sensitivity approximately doubles each time the number of variants tracked double (Supplementary Methods). The majority of cancer patients have greater than 48 somatic variants [57] and most patients in this study had close to this number tracked. The assay could have targeted greater than 48 variants in order to improve sensitivity further, however, an increasing number of patients would not have suitable numbers of variants and it would increase the risk of assay failure due to primer multiplexing. A fixed gene panel on the other hand may not be able to detect mutations in all patients, particularly in a heterogenous disease such as HNSCC that characteristically displays only few recurrent driver mutations, although where variants are present in fixed panels, this allows for rapid analysis, particularly where resources are limited. In the current cost-conscious healthcare environment, costs are an important consideration when determining assays that can be implemented in the clinic. Decreasing sequencing costs make the RaDaR^TM^ assay comparable to other platforms for similar applications, including large fixed NGS panels that are in widespread use.

In summary, findings from this prospective cohort study illustrate the potential of ctDNA as a biomarker of minimal residual disease detection and monitoring of early molecular-level recurrence in patients with HNSCC, demonstrating the feasibility of personalised ctDNA assays for therapy planning and follow-up. In this cohort, ctDNA was detected prior to surgery in 17 of 17 patients with HNSCC and during follow-up in all five patients who subsequently relapsed, with lead times ahead of clinical recurrence ranging from 108 to 253 days. In addition, although ctDNA levels will be displaying varying dynamics throughout the course of a patient’s management, personalised ctDNA monitoring could also be offered to patients primarily treated with non-surgical modalities. Early detection of relapse using ctDNA could therefore indicate patient populations where earlier therapeutic intervention may be beneficial. However, further interventional prospective studies with sufficient power are required before ctDNA analysis can be introduced into routine daily clinical practice.

## Supplementary information

Supplementary legends

Supp Methods

Suppl Figure 1

Supp Figure 2

Supp Table 1

Supp Table 2

Supp Table 3

## Data Availability

Data will be made available upon reasonable request to the submitting authors.
