# Supplementary material for: Liquid BIOpsy for MiNimal RESidual DiSease Detection in Head and Neck Squamous Cell Carcinoma (LIONESS)—a personalised circulating tumour DNA analysis in head and neck squamous cell carcinoma
Source: Br J Cancer. 2022 Feb 7;126(8):1186–95. doi: 10.1038/s41416-022-01716-7 (PMC9023460; doi:10.1038/s41416-022-01716-7)
Supplement: Supplementary file 1 — Supplementary legends [file 41416_2022_1716_MOESM1_ESM.docx]

**LIONESS: Legends to Supplementary Figures and Tables**

Supp Figure 1 – **Longitudinal monitoring of ctDNA for all patients in the study**

ctDNA detection is indicated with red circles, not detected (ND) with black circles for all plasma samples analysed. The blue box indicates start and end of adjuvant treatment, where applicable. Patient 9 had a second tumour recurrence which was deemed unresectable. Additional blood samples collected prior to the second clinically confirmed recurrence and death of the patient were positive for ctDNA (not analysed at time of manuscript submission). **Vertical line:** last clinical visit to date, **inverted yellow triangle:** clinical confirmation of disease relapse, **X:** documented death event.

Supp Figure 2 – **ctDNA detection in all patients who had a clinical recurrence**

Patients were stratified according to ctDNA detection at any time post-surgery (red curve) or no detection (black curve). Each vertical drop in the curve represents an event occurring, in this case relapse.

Supp Table 1: **Summary of the WES data performance characteristics**

**Key to columns:**

**Patient ID:** anonymised patient identifier

**reads (million):** total sequenced million reads

**aligned_pct:** percentage of aligned reads

**duplication_pct:** percentage of duplicated reads

**on_target_pct:** percentage of reads on target (within the capture region)

**usable_pct:** percentage of usable reads (i.e., aligned, on target and not duplicated)

**mean coverage:** mean read depth per nucleotide, based on usable reads

**total_variants_panel:** total number of variants designed for the primers panel

Supp Table 2: **Summary of variants designed as part of the personalised panels for each patient**

**Key to columns:**

**patient_id**: patient identifier

**mutation_code**: concise representation of the variant detected (e.g., mis_chr5_123456_A_T indicates a mismatch mutation, i.e. a SNV, at chromosome 5, position 123456 where an A to T mutation is identified)

**chr**: chromosome

**locus**: genomic position of the variant detected

**Gene.name**: gene where the variant belongs to

**Gene.description**: gene description based on the gencode v32 exons annotation

Supp Table 3: **ctDNA detection in plasma**

**Key to columns:**

**Patient ID:** anonymised patient identifier

**Sample_id**: sample identifier

**Input copies:** number of PCR amplifiable copies in each plasma sample

**eVAF**: estimated variant allele fraction. ND (not detected)

**mutant_molecules:** estimated number of mutant molecules

**mean_VAF:** mean measured variant allele frequency

**Score:** statistical score

**all_pass_variants:** number of variants passing all QC criteria per RaDaR panel

**total_variants:** total number of variants in the panel

**ctDNA_detected:** ctDNA detected

**accessionID:** accession identifier

**Timepoint:** description of the time point

Supp Methods: **RaDaR assay methods and analytical validation**
