# Supplementary material for: Liquid BIOpsy for MiNimal RESidual DiSease Detection in Head and Neck Squamous Cell Carcinoma (LIONESS)—a personalised circulating tumour DNA analysis in head and neck squamous cell carcinoma
Source: Br J Cancer. 2022 Feb 7;126(8):1186–95. doi: 10.1038/s41416-022-01716-7 (PMC9023460; doi:10.1038/s41416-022-01716-7)
Supplement: Supplementary file 2 — Supp Methods [file 41416_2022_1716_MOESM2_ESM.docx]

**RaDaR^TM^ assay methods and analytical validation**

The RaDaR^TM^ assay was analytically validated in Inivata’s CLIA laboratory (RTP, North Carolina) by trained operators. Personalised RaDaR^TM^ assays were designed and synthesised for 5 lung cancer patient samples and 2 breast cancer (HCC1395 & HCC1954) and 1 colon cancer (SW480) cell lines. The lung cancer patients were part of the INI-001 study [ClinicalTrials.gov identiﬁer: NCT02906852] and all patients had had ctDNA previously detected in their blood using InVisionFirst-Lung®.

To determine analytical specificity, the 8 personalised RaDaR^TM^ panels were tested against 69 blood samples from 18 healthy donors drawn into Streck cfDNA blood collection tubes. No calls were made in any of the 69 samples. A further 20 blood samples from 5 of the same healthy donors were drawn into EDTA tubes and run against the breast and colon cancer cell line RaDaR^TM^ assays and again no calls were made, thereby demonstrating 100% specificity (Table 1).

**Table 1:** Summary of the samples and panels used to determine RaDaR^TM^’s specificity

| **Specificity analysis** | | | | | |
| --- | --- | --- | --- | --- | --- |
| **Tube type** | Panel | Healthy donor blood tubes | Successfully tested | Detected | % Detected |
| **EDTA** | HCC1395 | 8 | 8 | 0 | 0% |
| **EDTA** | HCC1954 | 8 | 8 | 0 | 0% |
| **EDTA** | SW480 | 4 | 4 | 0 | 0% |
| **Streck** | HCC1395 | 20 | 20 | 0 | 0% |
| **Streck** | HCC1954 | 20 | 20 | 0 | 0% |
| **Streck** | SW480 | 9 | 9 | 0 | 0% |
| **Streck** | 5 NSCLC patients | 20 | 20 | 0 | 0% |

RaDaR^TM^’s Limit of Detection (LoD) was tested using the two breast cancer and one colon cancer cell lines. Cell line-derived DNA was diluted into normal DNA to produce contrived samples mimicking ctDNA at a range of levels near RaDaR^TM^’s target Limit of Detection (LoD). To achieve this, each of the cell lines along with normal DNA from the Genome in a Bottle sample NA12878 was sheared to approximately 160bp in length to mimic cfDNA fragmentation. NA12878 was selected as it has been extensively characterised with other sequencing methods. The fragmented DNA was quantified in copies per microliter by digital PCR (copies/µl) to enable accurate dilution. The cancer DNA was then serially diluted into normal DNA samples from 0.008%, 0.004%, 0.002% to 0.001% variant allele fraction (VAF) respectively.

Each cell line dilution was analysed in duplicate on 5 separate runs using 20,000 amplifiable copies of the genome as measured by digital PCR. The runs were performed by two operators with two reagent lots (Figure 1). In total, each dilution was assessed 30 times (10 replicates of each cell line). Sensitivity of RaDaR^TM^ was measured using Probit-based analysis. Using this approach, RaDaR^TM^’s LoD_95_ (the point 95% of samples are detected) is 0.0011% VAF. Good reproducibility was also observed between runs, operators, and reagent lots.


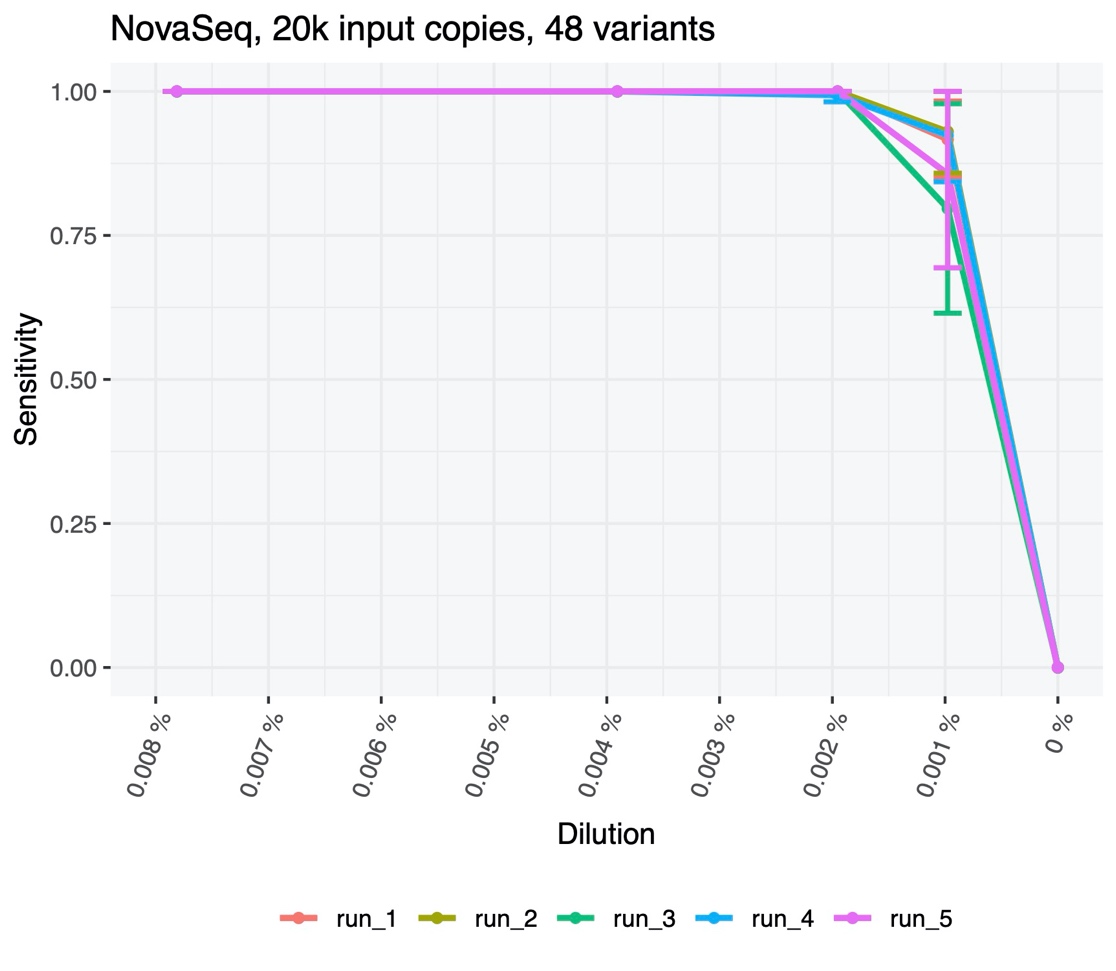


**Figure 1:** dilution-based analysis of RaDaR^TM^’s sensitivity using two breast cancer and one colon cancer cell line. Each line represents the percentage of detected samples when combining results across the cell lines in a single run.

To assess the impact the number of variants available has on the LoD, results from the cell line dilution study were randomly sub sampled (bootstrapped) removing different numbers of variants before calling. As the number of variants decreases, the sensitivity decreased in a predictable fashion (Table 2). As example, the LoD with 48 variants (0.0011%) is close to half the LoD when using half the number of variants (24 variant LoD_95_ = 0.0023%) This showed that targeting 48 variants improves RaDaR^TM^’s sensitivity. Furthermore, it demonstrated that in instances where less than 48 variants are available, the assay is still very sensitive, and this sensitivity is predictable based on the number of variants.

**Table 2:** Analysis of the impact the number of variants has on the LoD. For the 3 cell lines, variants were randomly removed before calling was performed and Probit was used to determine LoD with these sub-sampled data sets.

| **Number of variants** | **LoD_95_ VAF** |
| --- | --- |
| 48 | 0.00114 |
| 44 | 0.00128 |
| 40 | 0.00138 |
| 36 | 0.00152 |
| 32 | 0.00172 |
| 28 | 0.00190 |
| 24 | 0.00228 |
| 20 | 0.00276 |
| 16 | 0.00336 |
