# Supplementary material for: Liquid BIOpsy for MiNimal RESidual DiSease Detection in Head and Neck Squamous Cell Carcinoma (LIONESS)—a personalised circulating tumour DNA analysis in head and neck squamous cell carcinoma
Source: Br J Cancer. 2022 Feb 7;126(8):1186–95. doi: 10.1038/s41416-022-01716-7 (PMC9023460; doi:10.1038/s41416-022-01716-7)
Supplement: Supplementary file 3 — Suppl Figure 1 [file 41416_2022_1716_MOESM3_ESM.pdf]

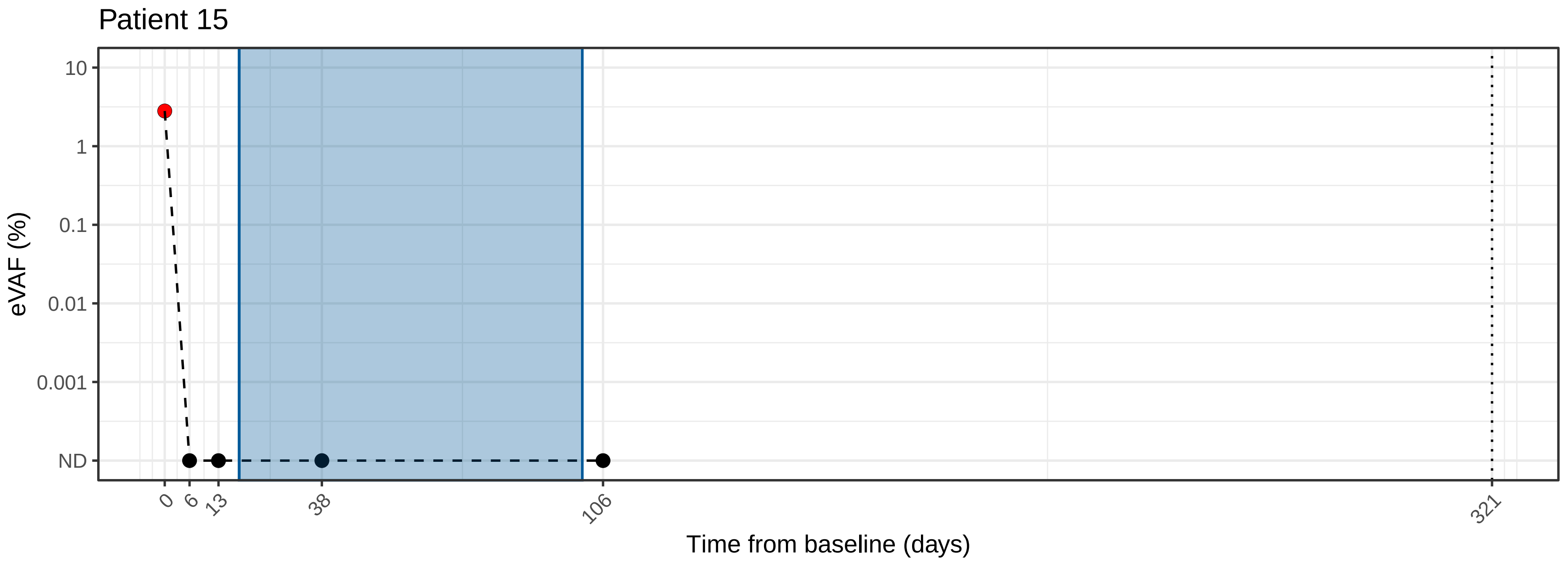

Patient 15
eVAF (%)
10
1
0.1
0.01
0.001
ND
0
6
13
38
106
321
Time from baseline (days)

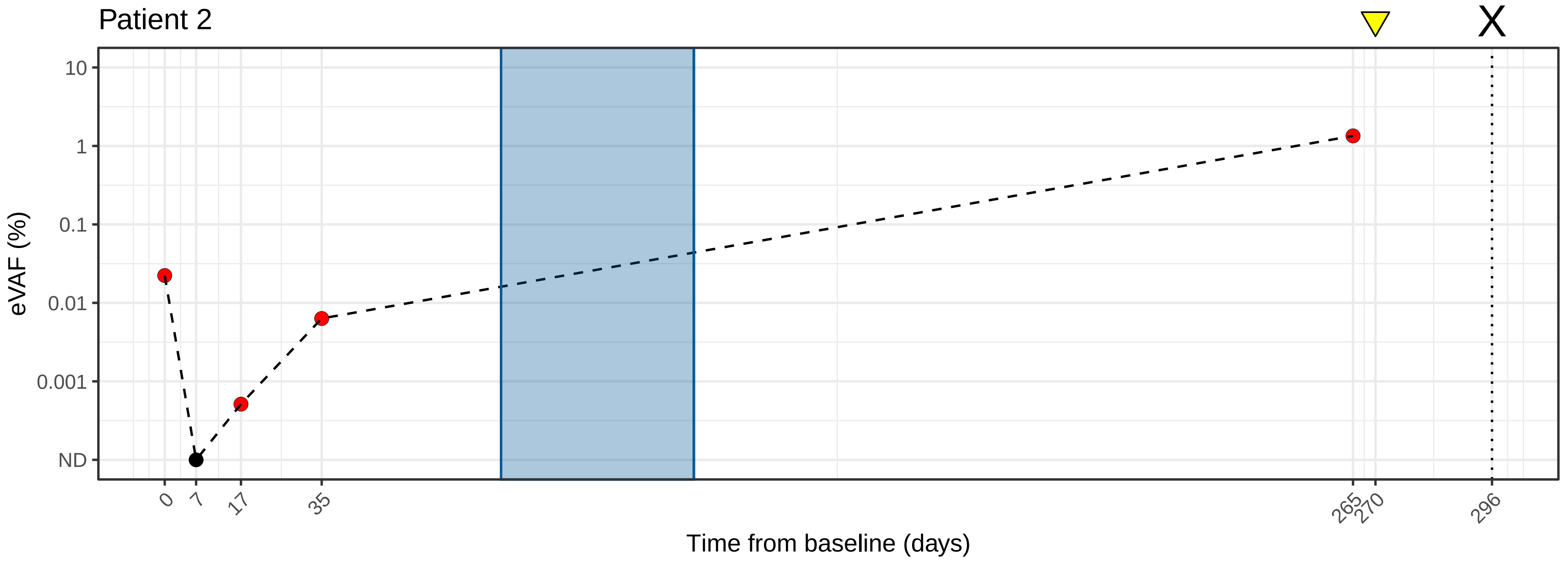
Patient 2
X
eVAF (%)
10
1
0.1
0.01
0.001
ND
0
7
17
35
265
270
296
Time from baseline (days)

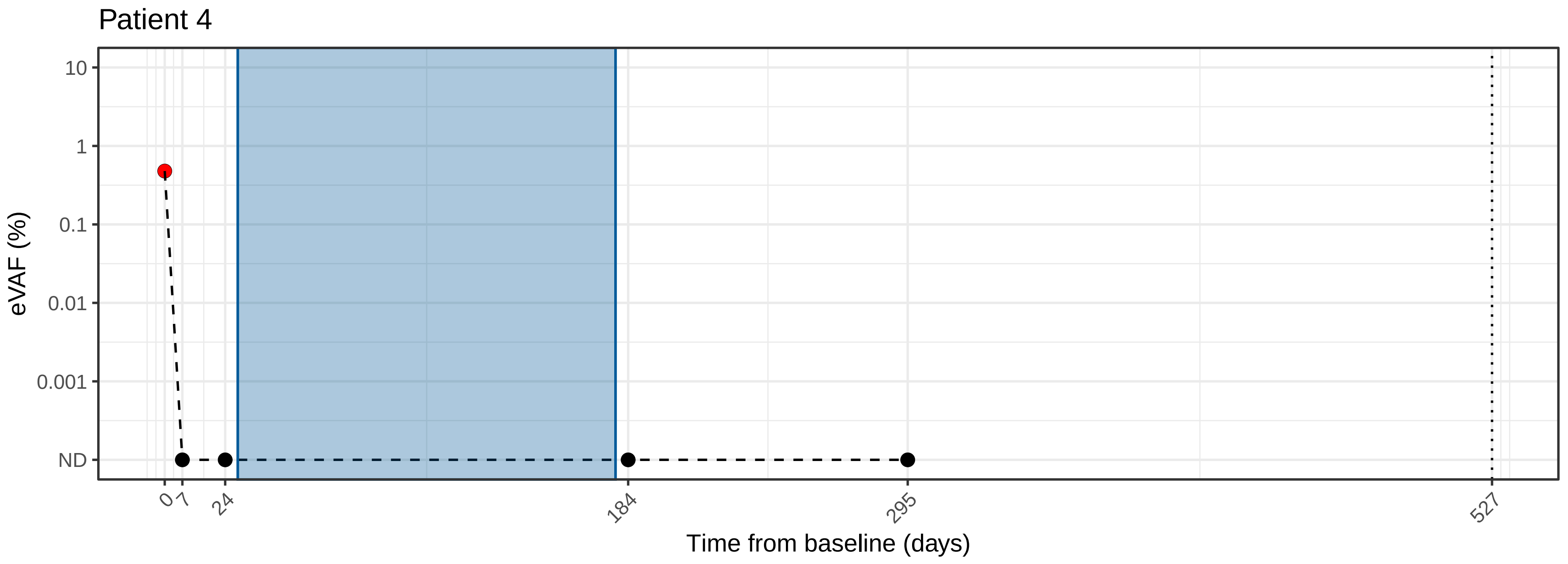

Patient 4
10
1
0.1
0.01
0.001
ND
eVAF (%)
0
7
24
184
295
527
Time from baseline (days)

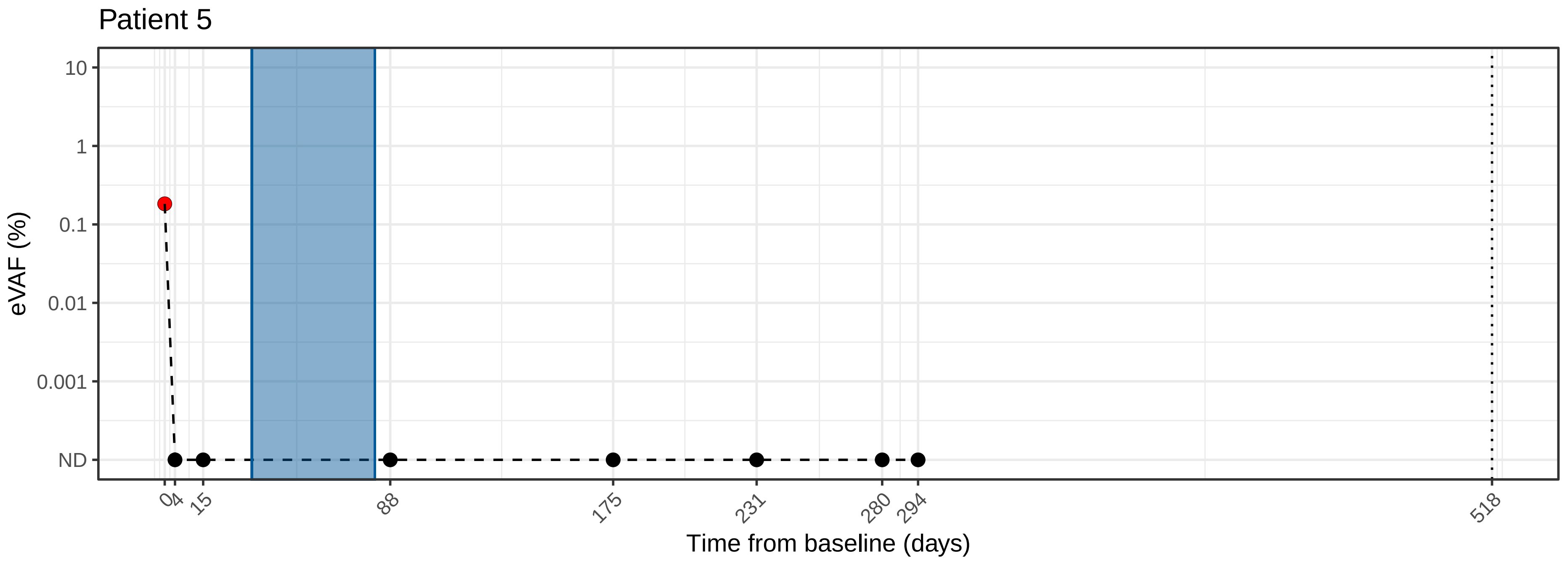
Patient 5
eVAF (%)
10
1
0.1
0.01
0.001
ND
0
4
15
88
175
231
280
294
518
Time from baseline (days)

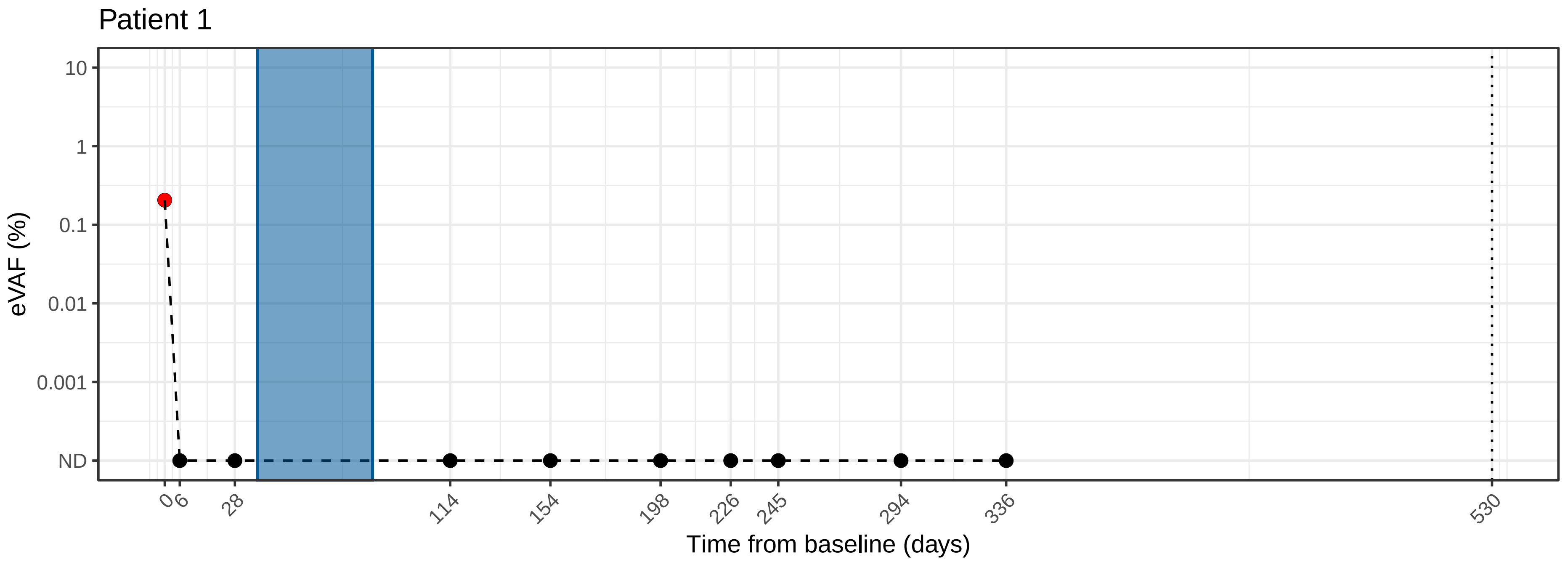

Patient 1
eVAF (%)
10
1
0.1
0.01
0.001
ND
0
6
28
114
154
198
226
245
294
336
530
Time from baseline (days)

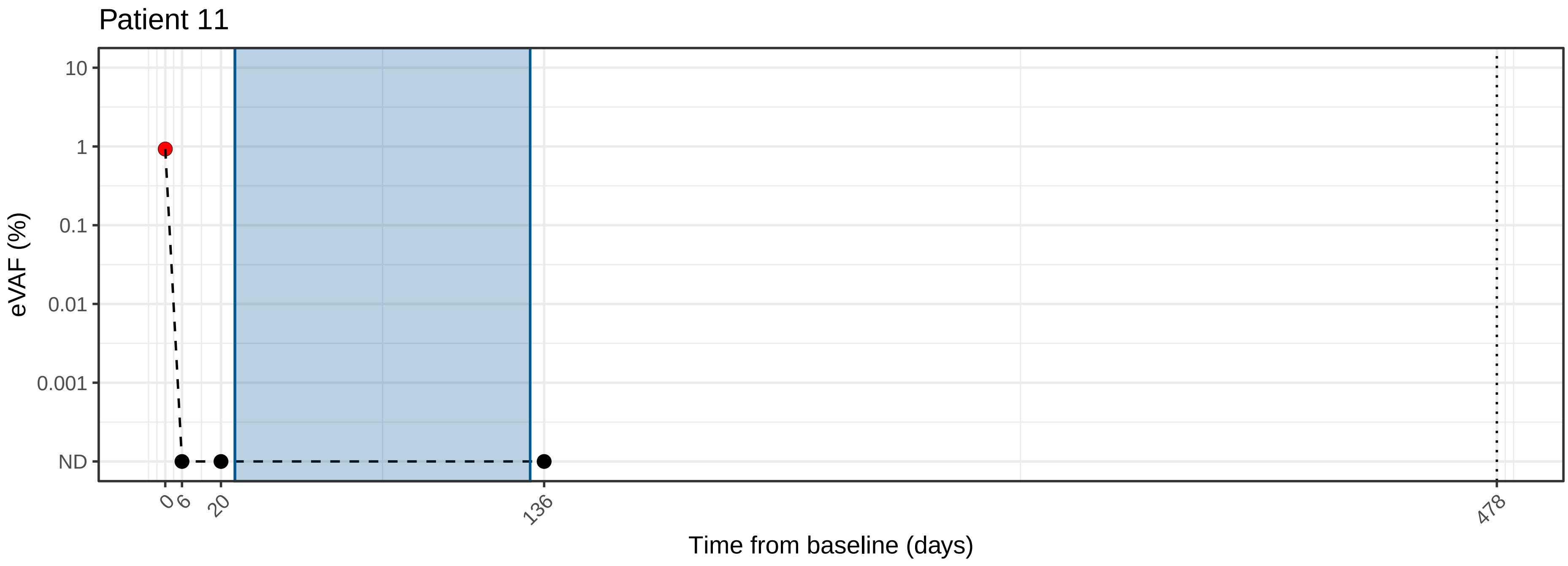

Patient 11
eVAF (%)
10
1
0.1
0.01
0.001
ND
0
6
20
136
478
Time from baseline (days)

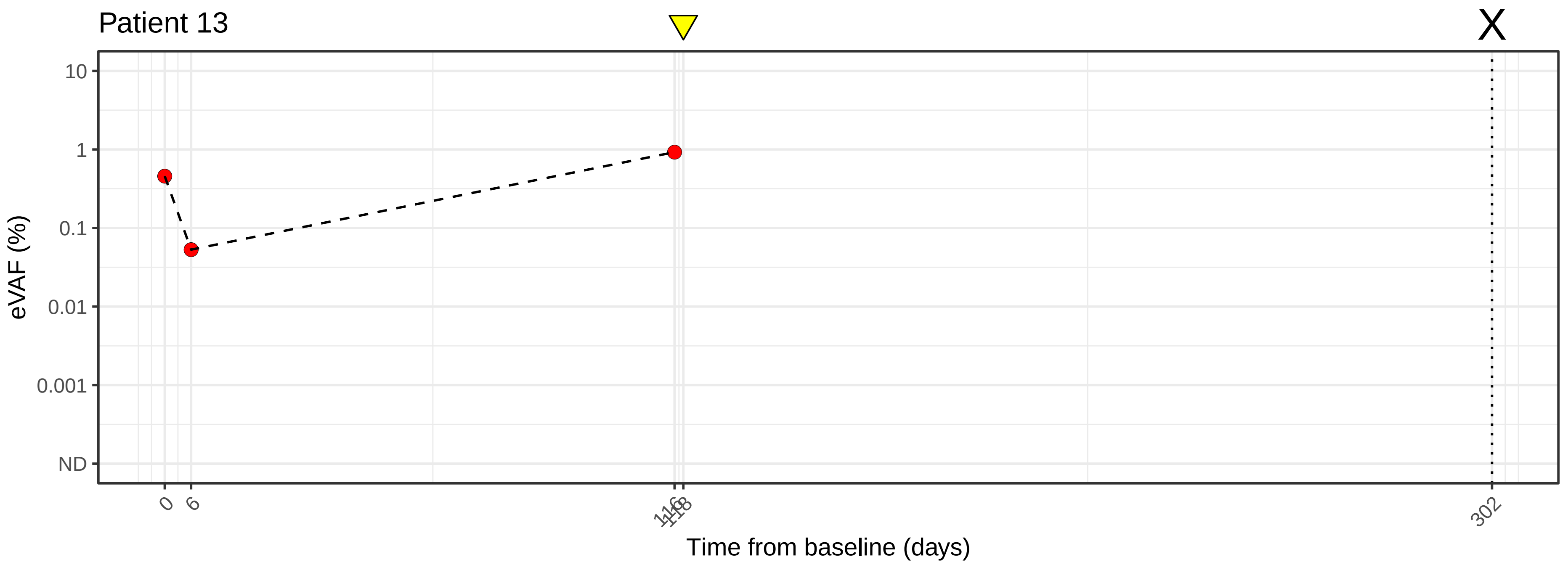

Patient 13
X
10
1
0.1
0.01
0.001
ND
eVAF (%)
0
6
116
118
302
Time from baseline (days)

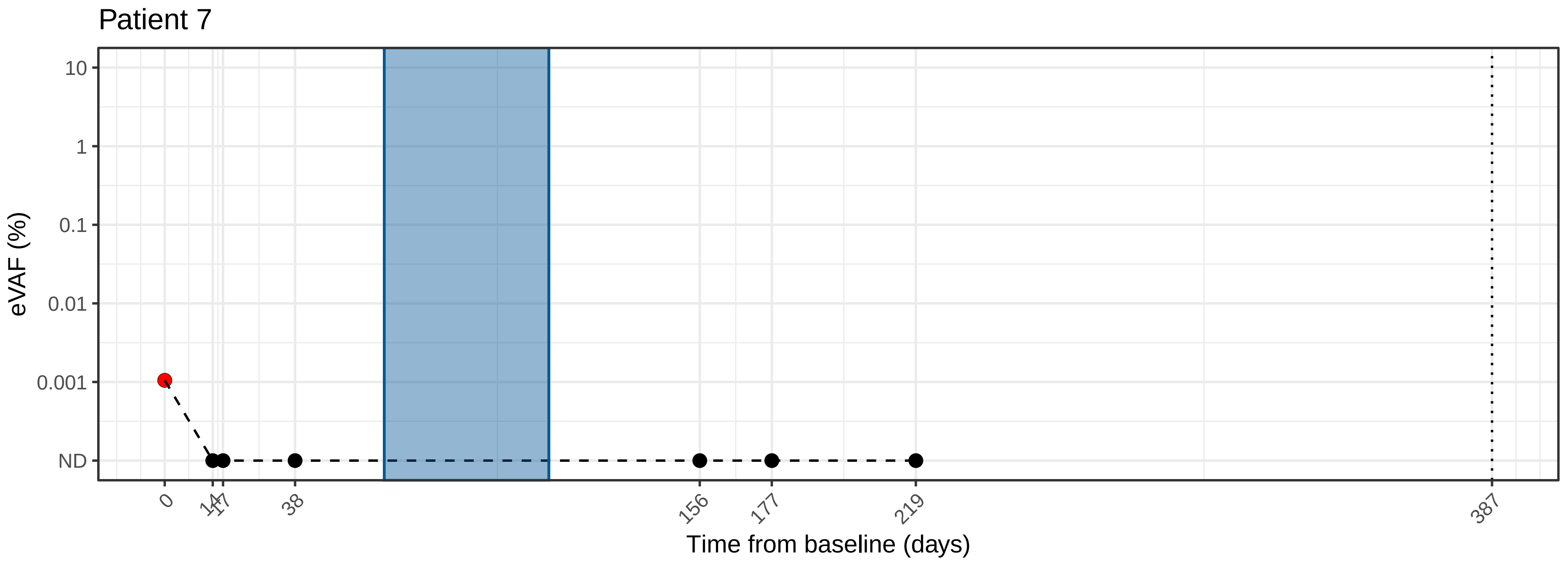

Patient 7
eVAF (%)
10
1
0.1
0.01
0.001
ND
0
14
17
38
156
177
219
387
Time from baseline (days)

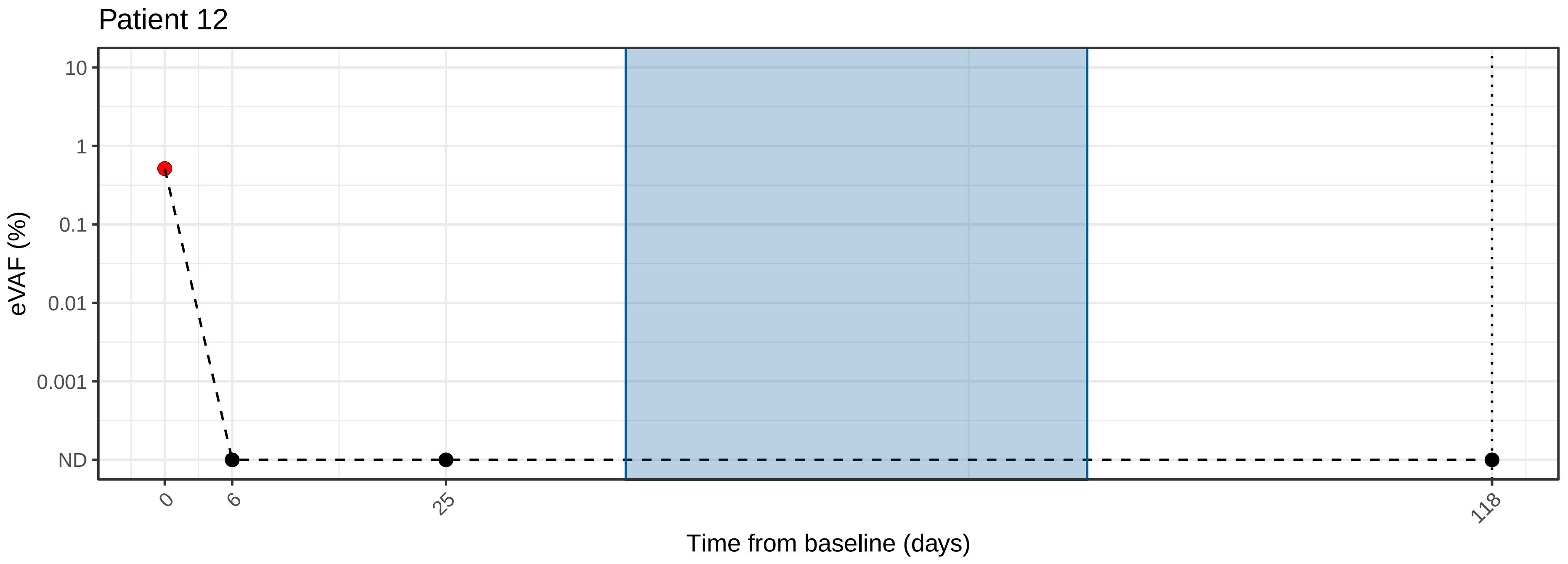
Patient 12
eVAF (%)
10
1
0.1
0.01
0.001
ND
0
6
25
118
Time from baseline (days)

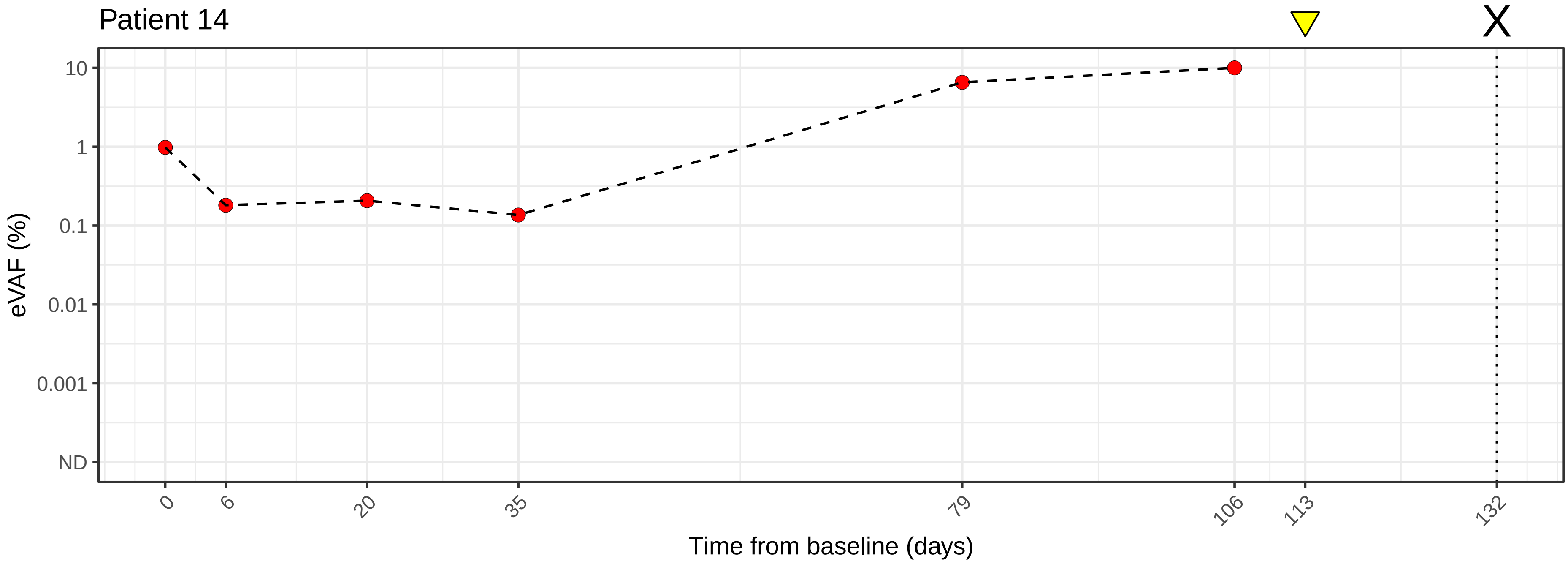

Patient 14
X
eVAF (%)
10
1
0.1
0.01
0.001
ND
0
6
20
35
79
106
113
132
Time from baseline (days)

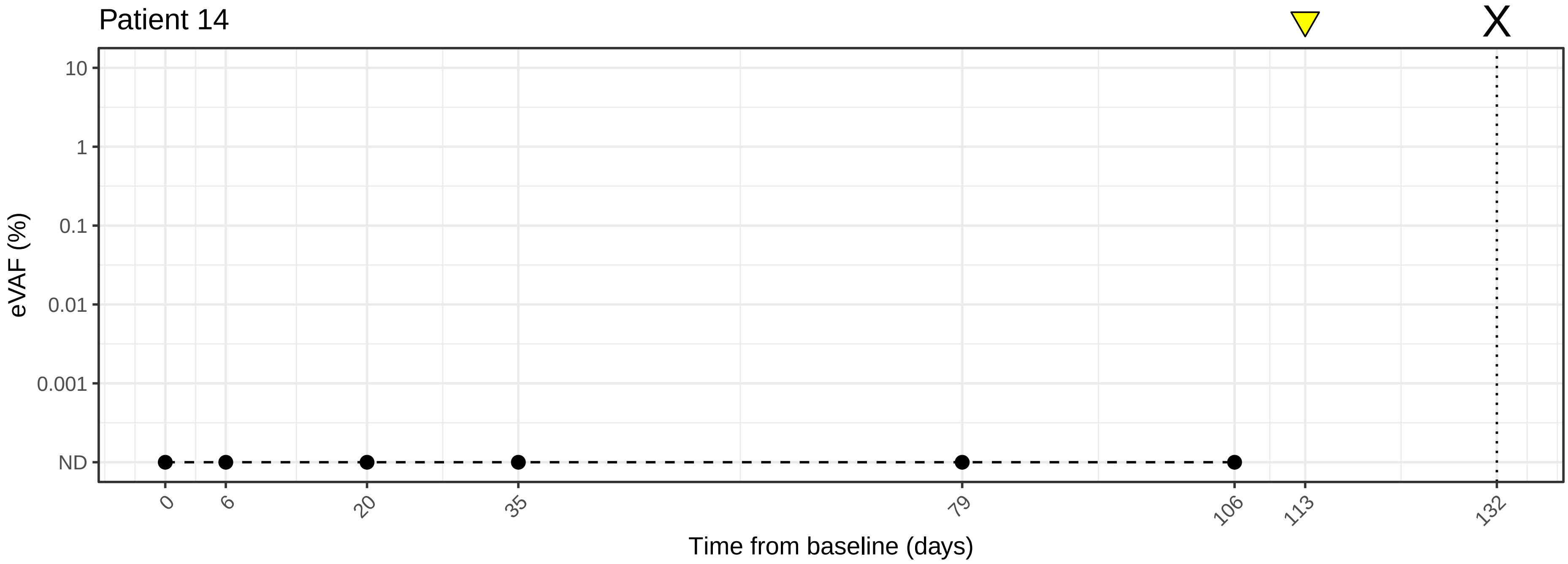

Patient 14
X
eVAF (%)
10
1
0.1
0.01
0.001
ND
0
6
20
35
79
106
113
132
Time from baseline (days)

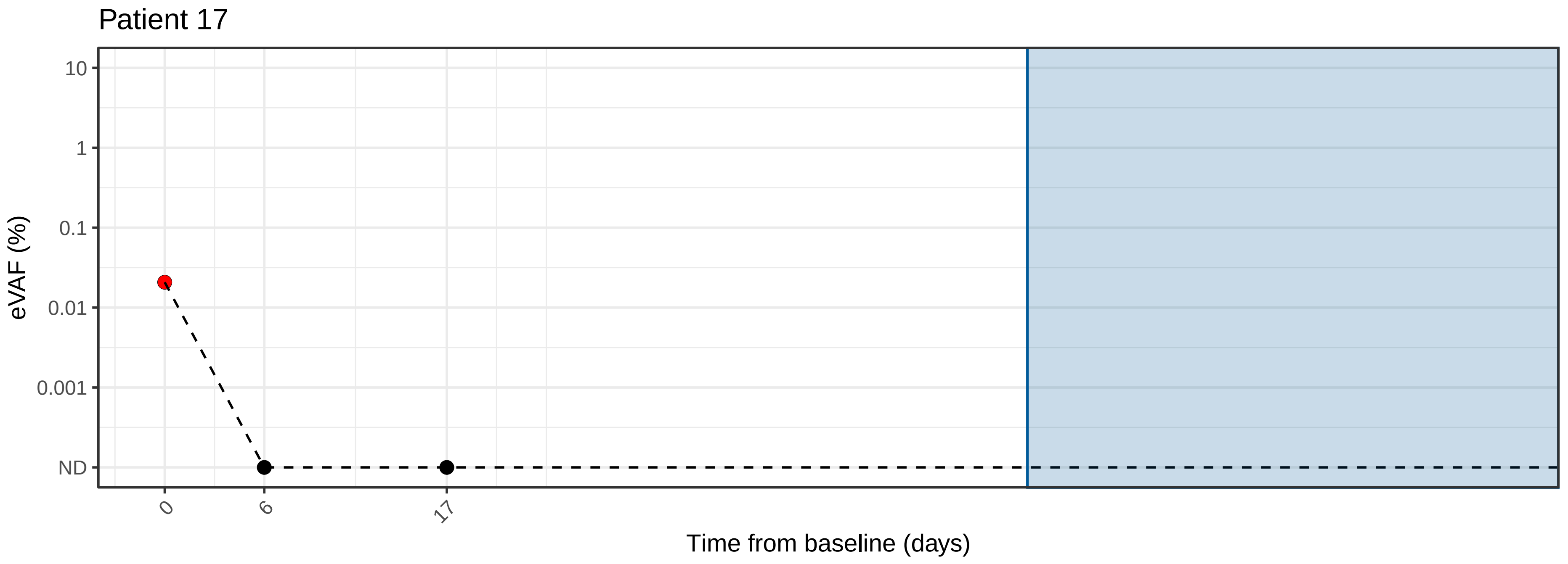

Patient 17
10
1
0.1
0.01
0.001
ND
eVAF (%)
0
6
17
Time from baseline (days)

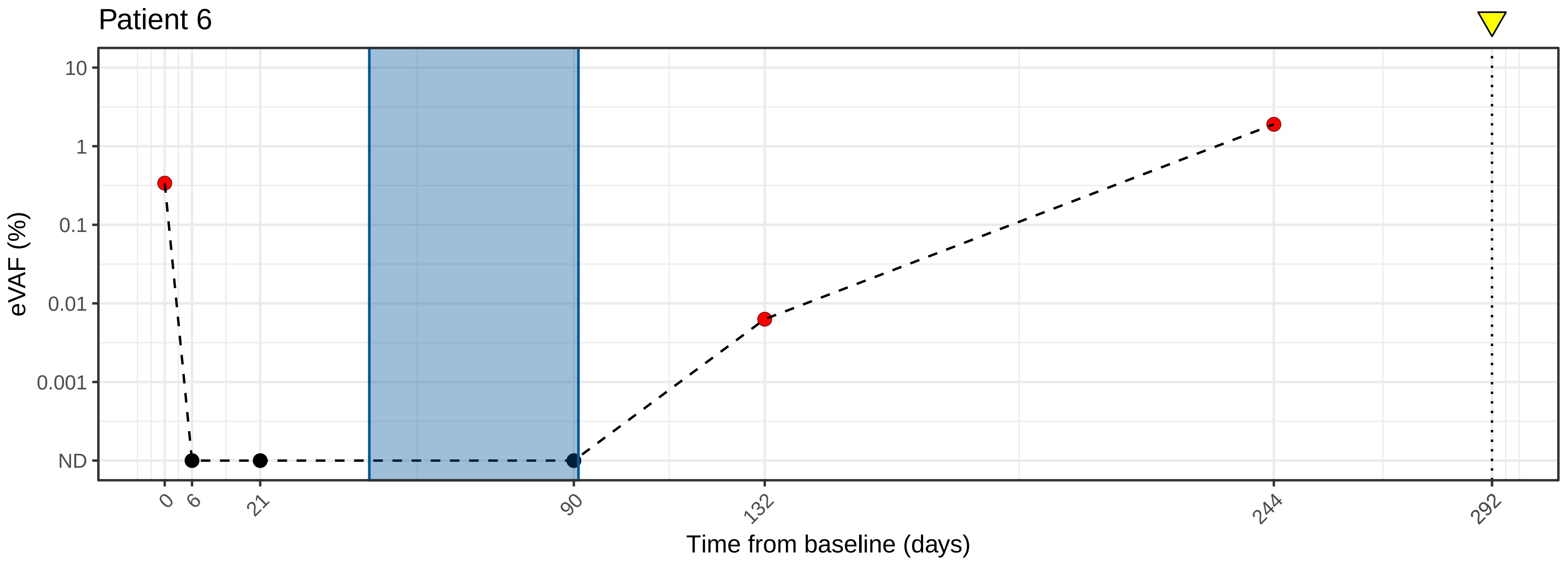

Patient 6
eVAF (%)
10
1
0.1
0.01
0.001
ND
0
6
21
90
132
244
292
Time from baseline (days)

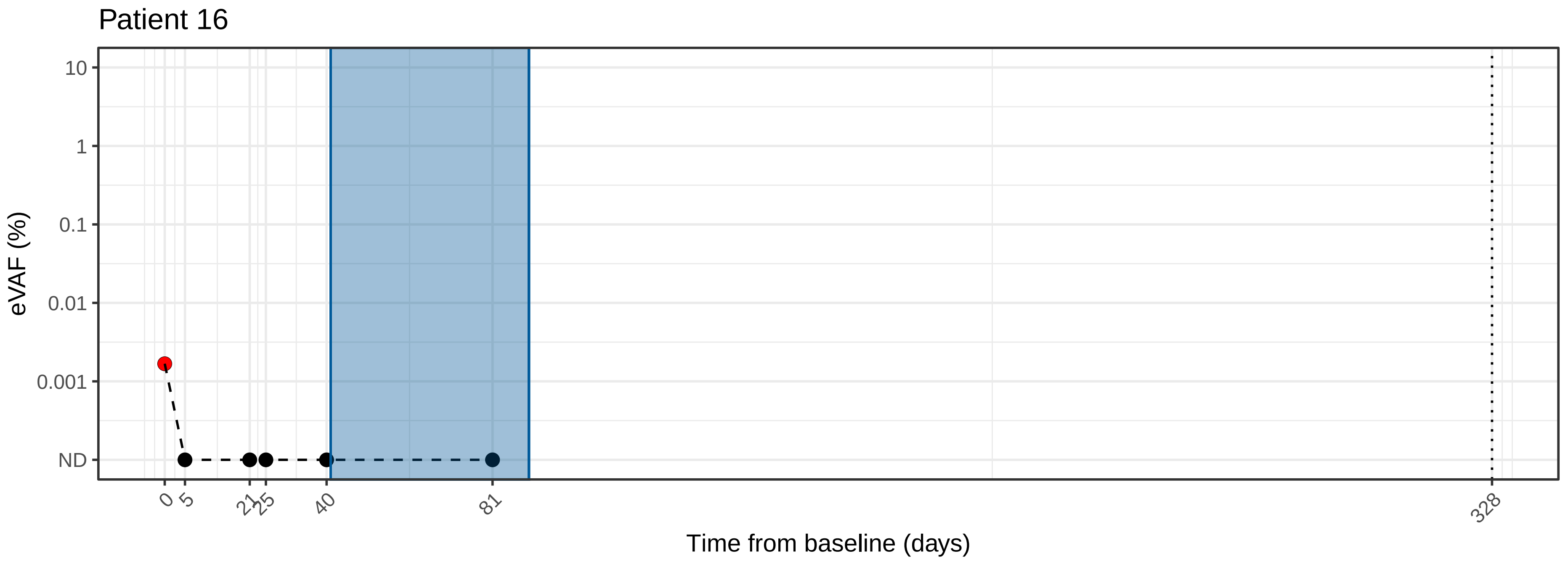

Patient 16
10
1
0.1
0.01
0.001
ND
eVAF (%)
0
5
21
25
40
81
328
Time from baseline (days)

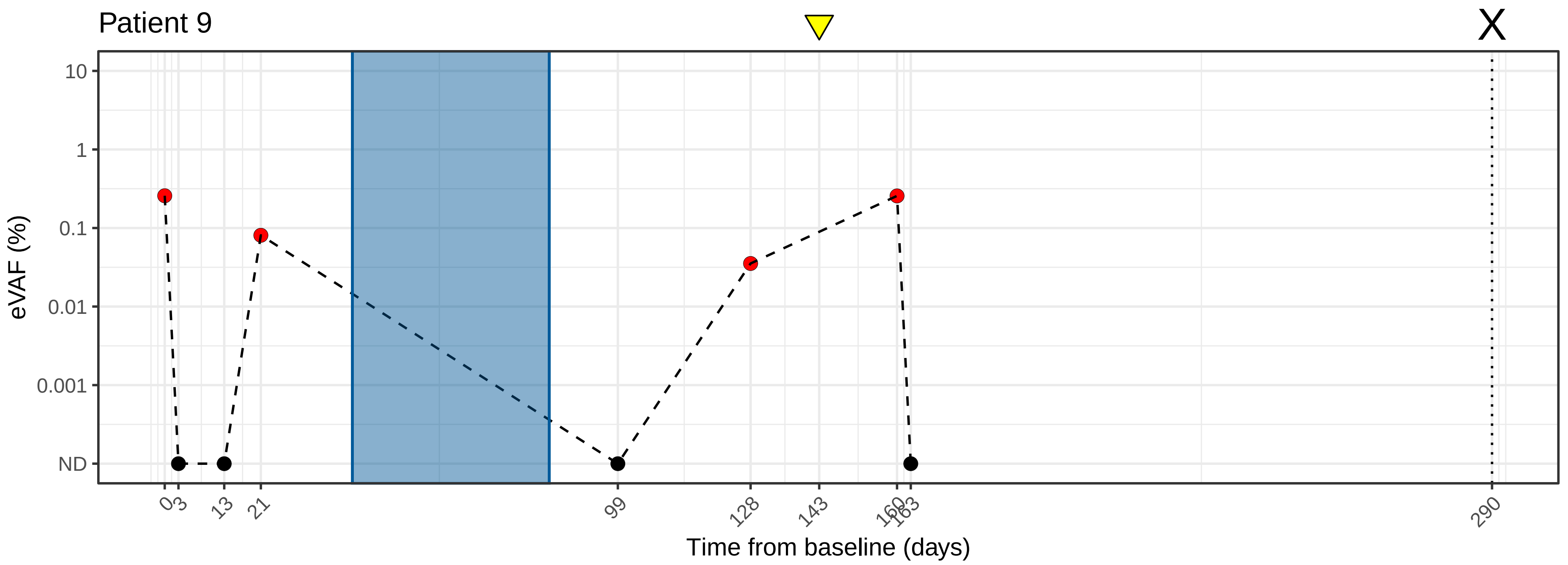
Patient 9
X
eVAF (%)
10
1
0.1
0.01
0.001
ND
0
3
13
21
99
128
143
160
163
290
Time from baseline (days)

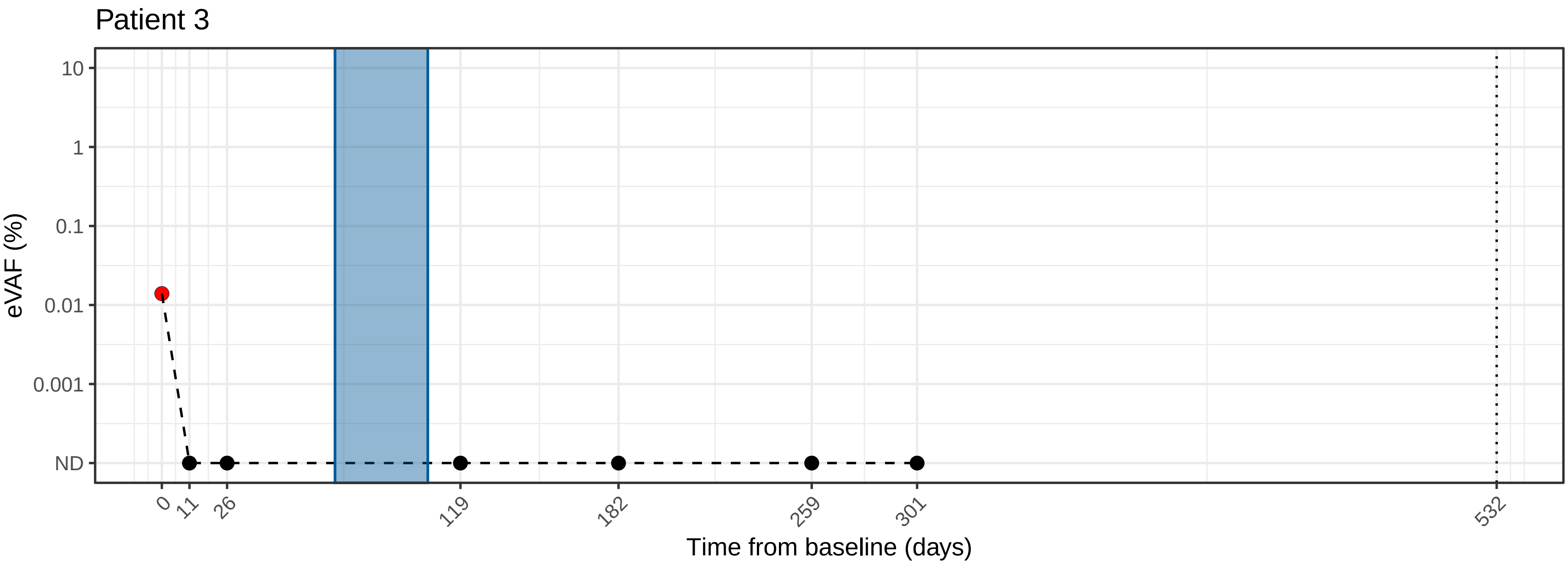
Patient 3
eVAF (%)
10
1
0.1
0.01
0.001
ND
0
11
26
119
182
259
301
532
Time from baseline (days)

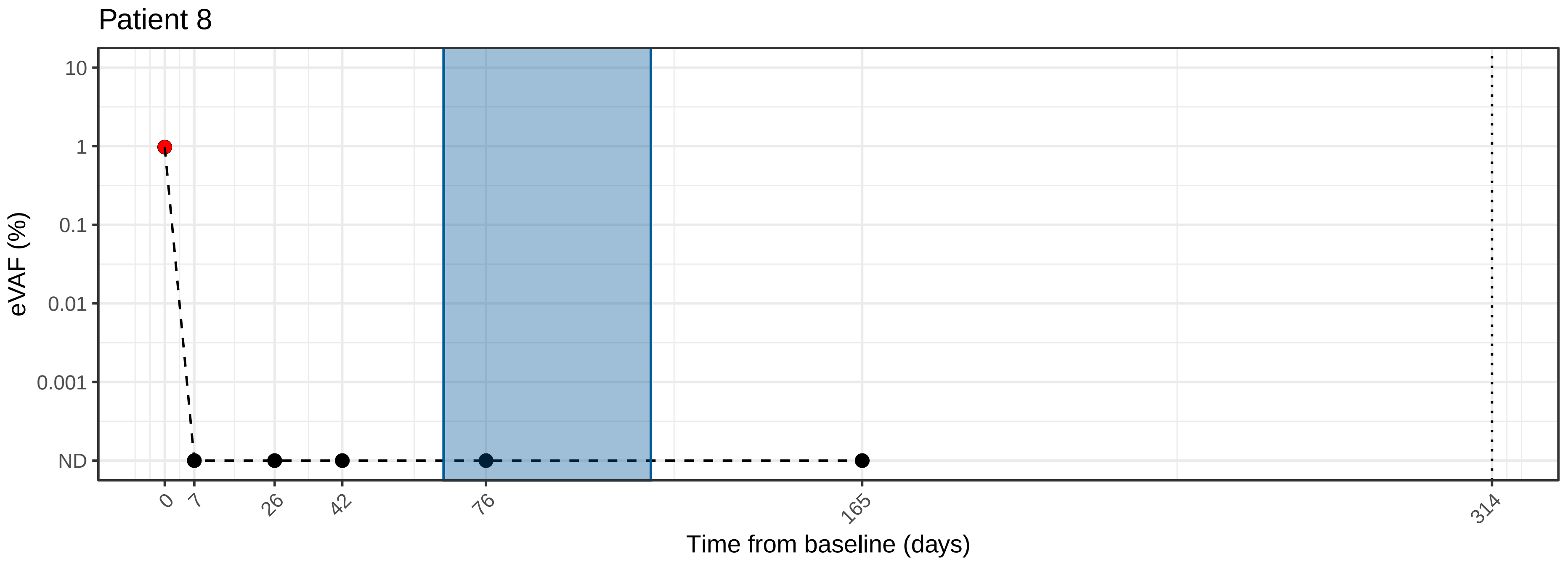

Patient 8
eVAF (%)
10
1
0.1
0.01
0.001
ND
0
7
26
42
76
165
314
Time from baseline (days)

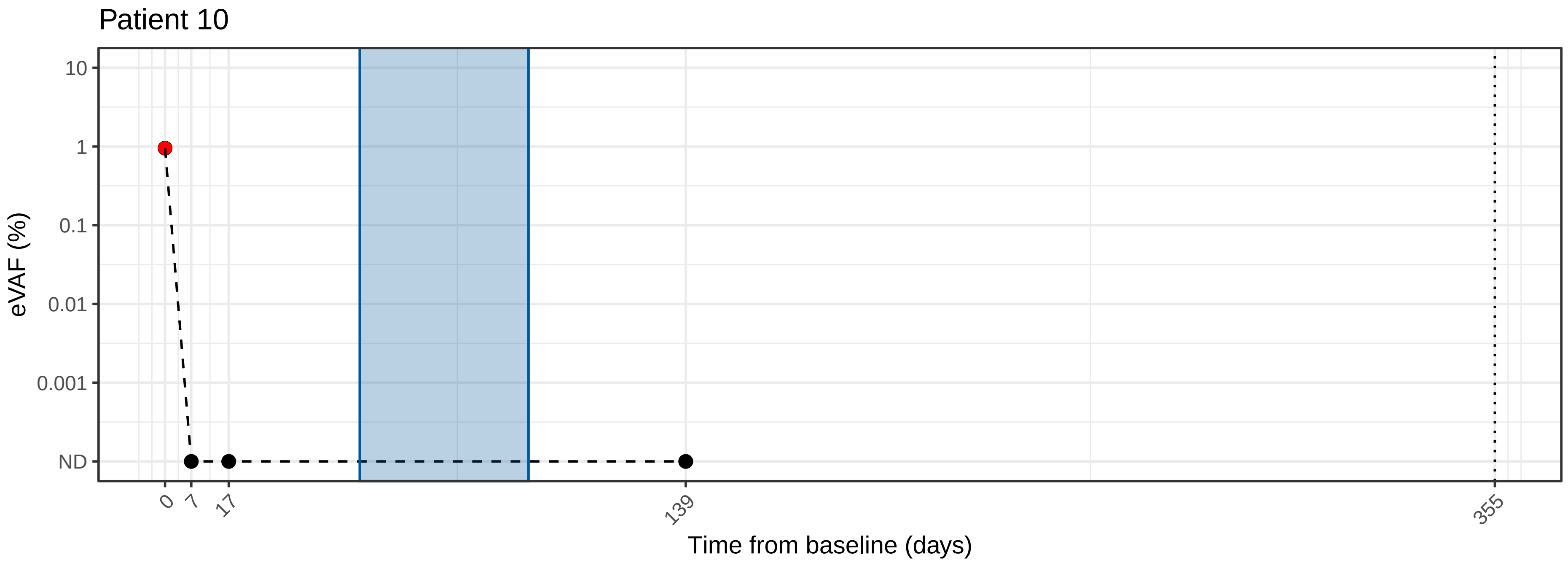
Patient 10
eVAF (%)
10
1
0.1
0.01
0.001
ND
0
7
17
139
355
Time from baseline (days)
