# Supplementary figures and images for: Liquid BIOpsy for MiNimal RESidual DiSease Detection in Head and Neck Squamous Cell Carcinoma (LIONESS)—a personalised circulating tumour DNA analysis in head and neck squamous cell carcinoma
Source: Br J Cancer. 2022 Feb 7;126(8):1186–95. doi: 10.1038/s41416-022-01716-7 (PMC9023460; doi:10.1038/s41416-022-01716-7)

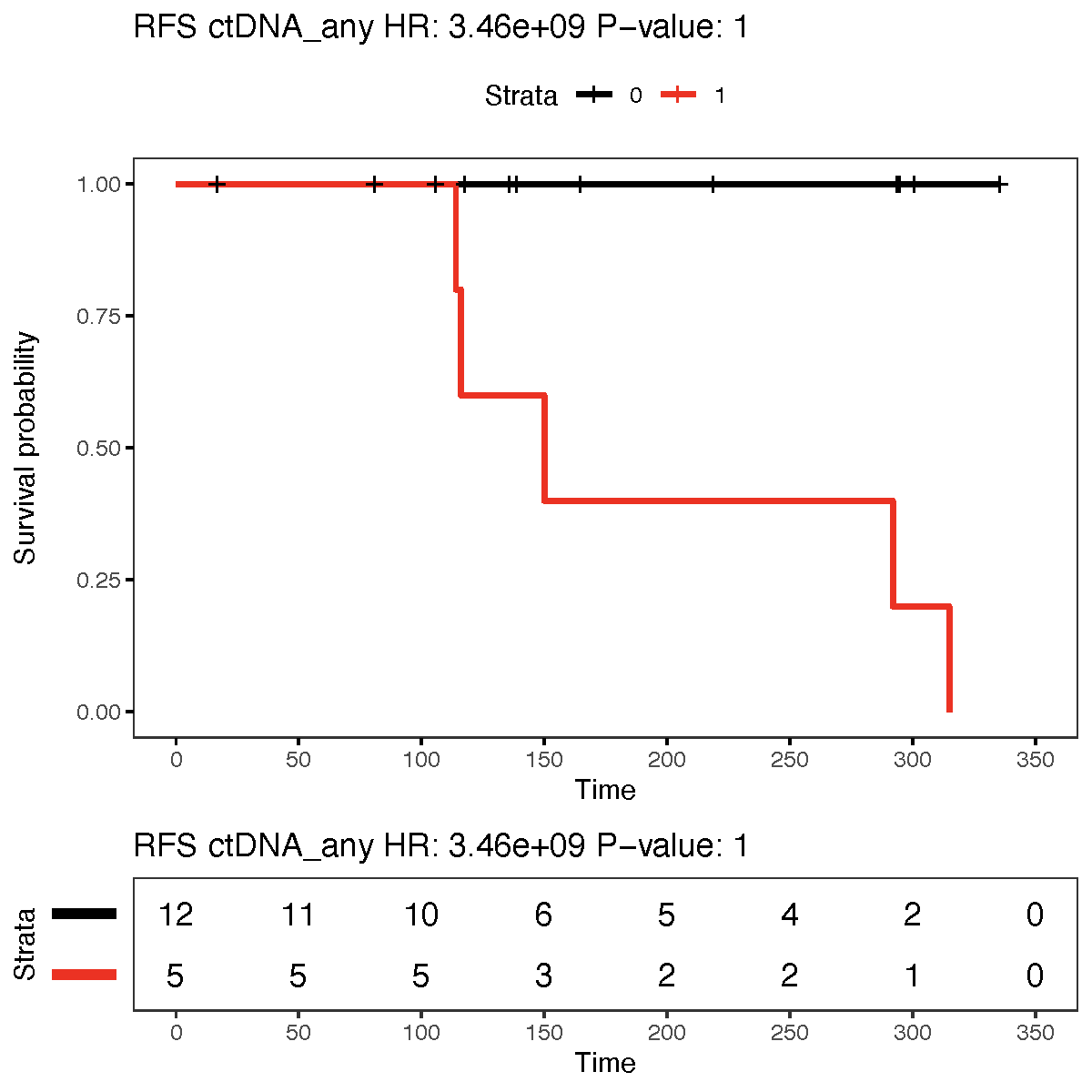

Supplement: Supplementary file 4 — Supp Figure 2 [file 41416_2022_1716_MOESM4_ESM.tif]
